# The Alkaloid-Enriched Fraction of *Pachysandra terminalis* (Buxaceae) Shows Prominent Activity against *Trypanosoma brucei rhodesiense*

**DOI:** 10.3390/molecules26030591

**Published:** 2021-01-23

**Authors:** Dagmar Flittner, Marcel Kaiser, Pascal Mäser, Norberto P. Lopes, Thomas J. Schmidt

**Affiliations:** 1 Institute of Pharmaceutical Biology and Phytochemistry (IPBP), University of Münster, PharmaCampus, Corrensstr. 48, D-48149 Münster, Germany; dagmar.flittner@web.de; 2 Swiss Tropical and Public Health Institute (Swiss TPH), Socinstrasse 57, CH-4051 Basel, Switzerland; marcel.kaiser@unibas.ch (M.K.); pascal.maeser@swisstph.ch (P.M.); 3 University of Basel, Petersplatz 1, CH-4003 Basel, Switzerland; 4 Núcleo de Pesquisa em Produtos Naturais e Sintéticos (NPPNS), Department of Biomolecular Sciences from School of Pharmaceutical Sciences of Ribeirão Preto, University of São Paulo, Av. Do Café s/n CEP, 14040-903 Ribeirão Preto-SP, Brazil; npelopes@fcfrp.usp.br

**Keywords:** *Pachysandra terminalis*, Buxaceae, steroid alkaloid, *Trypanosoma brucei*, mass spectrometry

## Abstract

In the course of our studies on antiprotozoal natural products and following our recent discovery that certain aminosteroids and aminocycloartanoid compounds from *Holarrhena africana* A. DC. (Apocynaceae) and *Buxus sempervirens* L. (Buxaceae), respectively, are strong and selective antitrypanosomal agents, we have extended these studies to another plant, related to the latter—namely, *Pachysandra terminalis* Sieb. and Zucc. (Buxaceae). This species is known to contain aminosteroids similar to those of *Holarrhena* and structurally related to the aminocycloartanoids of *Buxus*. The dicholoromethane extract obtained from aerial parts of *P. terminalis* and, in particular, its alkaloid fraction obtained by acid–base partitioning showed prominent activity against *Trypanosoma brucei rhodesiense (Tbr)*. Activity-guided fractionation along with extended UHPLC-(+)ESI QTOF MS analyses coupled with partial least squares (PLS) regression modelling relating the analytical profiles of various fractions with their bioactivity against *Tbr* highlighted eighteen constituents likely responsible for the antitrypanosomal activity. Detailed analysis of their (+)ESI mass spectral fragmentation allowed identification of four known constituents of *P. terminalis* as well as structural characterization of ten further amino-/amidosteroids not previously reported from this plant.

## 1. Introduction

Human African Typanosomiasis (HAT or “sleeping sickness”) is a neglected tropical disease (NTD) caused by the kinetoplastid parasites *Trypanosoma brucei* gambiense and *T. b. rhodesiense* [1]. Without treatment, this disease is inevitably fatal. As the few existing medications are toxic or difficult to administer, the search for new active chemical entities (CEs) against African trypanosomes remains an urgent goal. In spite of recent success with the introduction of the first orally active antitrypanosomal drug, Fexinidazole [2], it remains important to add new CEs with possibly new mechanisms of action to the drug development pipeline against *T. brucei*.

In previous studies, we have discovered that certain aminosteroid alkaloids from an African Apocynacea, *Holarrhena africana* A. DC. (a synonym of the more commonly used *Holarrhena floribunda* T. Durand and Schinz), are very potent and highly selective trypanocides [3,4,5]. Similarly, an aminocycloartanoid alkaloid, Cyclovirobuxein B from *Buxus sempervirens* L. (Buxaceae), structurally related to the compounds from *Holarrhena*, was found to be strongly active and selective against this parasite [4]. We have therefore extended our study to another widespread ornamental plant from the family Buxaceae, *Pachysandra terminalis* Sieb. and Zucc., which is also known to contain a wide variety of amionosteroids [6,7]. Some bioactivities have been described for *Pachysandra* alkaloids, in particular cytotoxic and antitumoral effects [6,7]. No account of its potential antitrypanosomal activity exists. In this communication, we wish to report on the antiprotozoal activity of the crude extracts, the alkaloid-enriched fraction and a variety of fractions of this plant, along with detailed analyses of their UHPLC-(+)ESI MS profiles, which give first hints at the active constituents.

## 2. Results and Discussion

### 2.1. Crude Extract, Alkaloid-Enriched Fraction and Their Antitrypanosomal Activities

The dichloromethane (DCM) extract (PS_DCM) obtained from the aerial parts of *P. terminalis* was tested initially for in vitro activity against *Trypanosoma brucei rhodesiense* (bloodstream forms; *Tbr*), *T. cruzi* (intracellular amastigotes), *Leishmania donovani* (axenic amastigotes) and *Plasmodium falciparum* (intraerythrocytic forms). Interestingly, the crude extract turned out to have selective activity against *Tbr* (see Table 1), as opposed to that of *B. sempervirens,* whose extract presented selective antiplasmodial activity in our earlier study [8].

Therefore, its alkaloid-enriched fraction (PS_AEF) obtained by acid/base partitioning, as well as the nonbasic neutral fraction (PS_NF), was also tested against the parasites. PS_AEF turned out to contain the active principle, since it was more than ten times more active than the crude extract against *Tbr*, while the PS_NF was much less active (see Table 1). PS_AEF also showed some activity against *Pf*, but further work concentrated on *Tbr* alone. PS_AEF also showed considerable selectivity towards Tbr when compared to mammalian control cells (L6 rat skeletal myoblasts) with a selectivity index of 24 (ratio of IC_50_ L6/IC_50_ *Tbr*).

**Figure 1:**
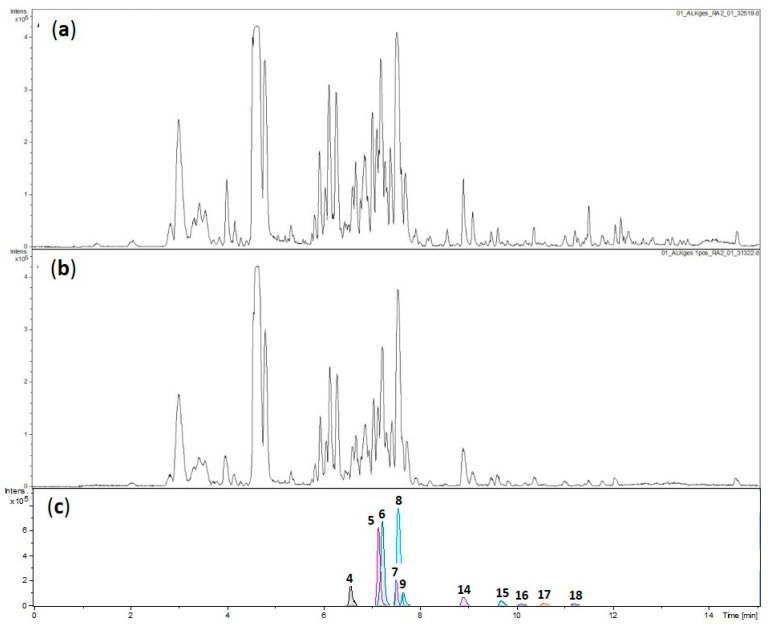
LC–MS profiles (base peak chromatograms of *m/z* 50–1500) of PS_AEF (**a**) obtained from a small-scale extract and (**b**) from a larger scale extract of aerial parts of *P. terminalis*. Constituents highlighted by the partial least squares (PLS) model (compare Figure 2 and Table 2) are shown as dissect compound chromatograms in (**c**), obtained from the analysis shown in (**b**). Note that some of the compounds were too low in concentration to be detected at the crude extract level.

Since the extraction at this stage had been performed with a small amount of plant material (27 g), it was repeated at a larger scale (2.6 kg) for a more detailed investigation. Even though the activity of the PS_AEF from the larger scale extraction was somewhat lower than before (IC_50_ against *Tbr* of 3.6 µg/mL), this preparation was further fractionated since its UHPLC-(+)ESI MS profile (henceforth termed LC–MS) was essentially identical to that from the first extraction (see Figure 1). These LC–MS analyses show the great complexity of the alkaloid mixture in the total PS_AEF.

### 2.2. Fractionation, Bioactivity Testing and Localization of Presumably Active Constituents by UHPLC–MS Profiling and PLS Regression Modelling

**Figure 2:**
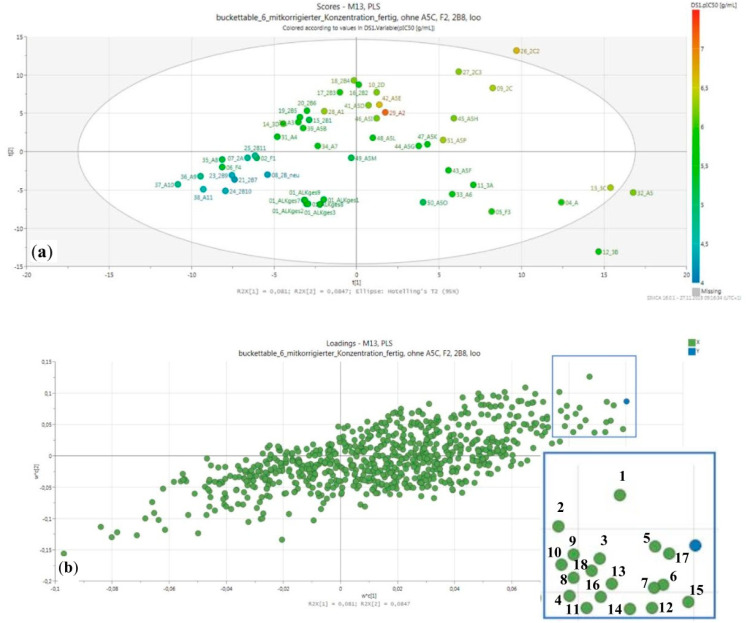
(**a**) Scores plot and (**b**) loadings plot of the second vs. the first PLS component of the PLS regression model. The selected most important variables (=buckets) are marked with a square in the loadings plot; the same region is shown as an enlarged inset with the bucket numbers (compare Table 2).

The PS_AEF was therefore fractionated by Column chromatography (CC) on silica, followed by various other methods (see Figure 8, Material and Methods section) into a variety of fractions and subfractions, most of which were then tested for activity against Tbr. IC_50_ values are reported in Figure 8, Section 3.3. The results indicated that the activity did not concentrate in a particular part of the fractionation scheme so that various constituents in different fractions should account for the strong overall activity. However, the more active samples were still rather complex mixtures of various alkaloids as indicated by the LC–MS analyses of all fractions. Therefore, an attempt was made to obtain hints at the main constituents responsible for the activity by using multivariate statistics. To this end, the LC–MS profiles were transformed into a bucket table using the Profile Analysis software (v. 2.1, Bruker Daltonics, Bremen, Germany) and then analyzed by partial least squares regression (PLS) as implemented in SIMCA (v. 16.0.1, Sartorius stedim biotech, Umeå, Sweden) using the respective fractions’ IC_50_ values as the dependent variable. A final PLS model explaining about 95% of the variance in the biological data was obtained for 55 samples, which consisted of four significant latent variables (PLS components). The model yielded reasonable statistical quality with a coefficient of determination (R^2^) of 0.95 and a coefficient of determination after leaving one out cross-validation (Q^2^) of 0.66. The resulting scores and loadings plots of the second vs. the first PLS component are shown in Figure 2. From the loadings plot, the 18 most influential variables (i.e., those with highest weights on the first (and high weights also on the second) PLS component, marked with a square in Figure 2b) were selected and the corresponding mass spectra were inspected. The compounds represented by these variables are listed in Table 2 with their MS characteristics.

### 2.3. Structural Characterization of Presumably Active Constituents Based on Their +ESI QTOF Mass Spectra

Of the 18 compounds selected from the PLS regression model, four could readily be dereplicated by matching their +ESI QTOF mass spectral data with the literature whereas the structures of most others could be assigned on grounds of thorough analysis of their mass spectra (MS^2^ spectra and fragmentation pathways are shown as Figures S1–S18, Supplementary Material).

Thus, compound **8** (bucket 7.58:451; note that bucket characteristics *tR*:*m/z* of [M + H]^+^ will be mentioned in parenthesis in the following descriptions; only nominal *m/z* values are used in the main text; exact masses are reported Figures S1–S18, Supplementary Material) was identified as Epipachysamine D, a known constituent of *P. terminalis* [9,10]. Its mass spectrum (Figure S8, Supplementary Material) was in agreement with the reported data for this aminosteroid [9]. However, new mechanistic explanations are presented as the steroid skeletons tend to form charge retention fragmentations, as recently revised in the case of some steroidal alkaloids [11]. Likewise, compounds **1** (6.21:344), **3** (6.72:3.28) and **6** (7.24:5.23) could be assigned by comparison of their MS data (Figures S1, S3 and S6, Supplementary Material) with the literature on known constituents of *P. terminalis* as *E/Z* salignone [7,12], 3β-Methylamino-16-oxo-5,17(20)-*cis/trans*-pregnadiene [7] and Pachysandrine A [13], respectively.

**Table 2:** Mass spectral characteristics of 18 presumably important compounds selected from the PLS regression model (compare Figure 2).

Comp.	Bucket [*tR*:*m/z*]	[M + H]^+^ Elem. Formula	Prominent Fragments	Identity Assignment
**1**	6.21:344.289	C_23_H_38_NO	299;**281**;107	*E/Z-*Salignone ^1^
**2**	6.63:222.151	n.a.		n.a.
**3**	6.72:328.263	C_22_H_34_NO	297;**279**;119;105	3β-Methylamino-16-oxo-5,17(20)-*cis/trans*-pregnadiene ^1^
**4**	6.82:481.355	C_31_H_49_N_2_O_2_	436;314;**283**;105	see Figure 3 ^3^
**5**	7.15:509.375	C_32_H_49_N_2_O_3_	418;**283**;314;105	see Figure 3 ^3^
**6**	7.24:523.392	C_33_H_51_N_2_O_3_	478;436;418;**283**;136;105	Pachysandrin A ^1^
**7**	7.57:449.340	C_30_H_45_N_2_O	418;387;122;105	see Figure 3 ^3^
**8**	7.58:451.368	C_30_H_47_N_2_O	406;**285**;122;105	Epipachysamine D ^2^
**9**	8.02:455.385	C_29_H_47_N_2_O_2_	424;406;314;**279**;136;122;111	see Figure 3 ^3^
**10**	9.22:523.356	C_32_H_47_N_2_O_4_	463;441;**283**,136;105	see Figure 3 ^3^
**11**	9.72:469.345	C_29_H_45_N_2_O_3_	387;359;328;300	n.a.
**12**	9.79:441.346	C_28_H_45_N_2_O_2_	359;328;**283**;121;100;86;83	see Figure 3 ^3^
**13**	9.80:471.360	C_29_H_47_N_2_O_3_	387;359;328;300	n.a.
**14**	9.88:515.386	C_31_H_51_N_2_O_4_	455;433;373;314;**283**;114;86	see Figure 3 ^3^
**15**	9.99:537.370	C_33_H_49_N_2_O_4_	477;455;342;**283**;136,105;86	see Figure 3 ^3^
**16**	10.10:443.365	C_28_H_47_N_2_O_2_	443;384;**285**;100;86;83	see Figure 3 ^3^
**17**	10.62:477.336	C_31_H_45_N_2_O_2_	477;418;392;172;86	n.a.
**18**	11.18:412.319	C_27_H_42_NO_2_	386;313;**281**;157;131;100;83	see Figure 3 ^3^

^1^ Dereplicated as known constituents of *P. terminalis* on grounds of known fragmentation characteristics as described in [9]. ^2^ Dereplicated by direct match of MS data with literature. ^3^ Structure proposed on grounds of known fragmentation characteristics as described in [9]; compare Figure 4. n.a.: not assigned.

**Figure 4:**
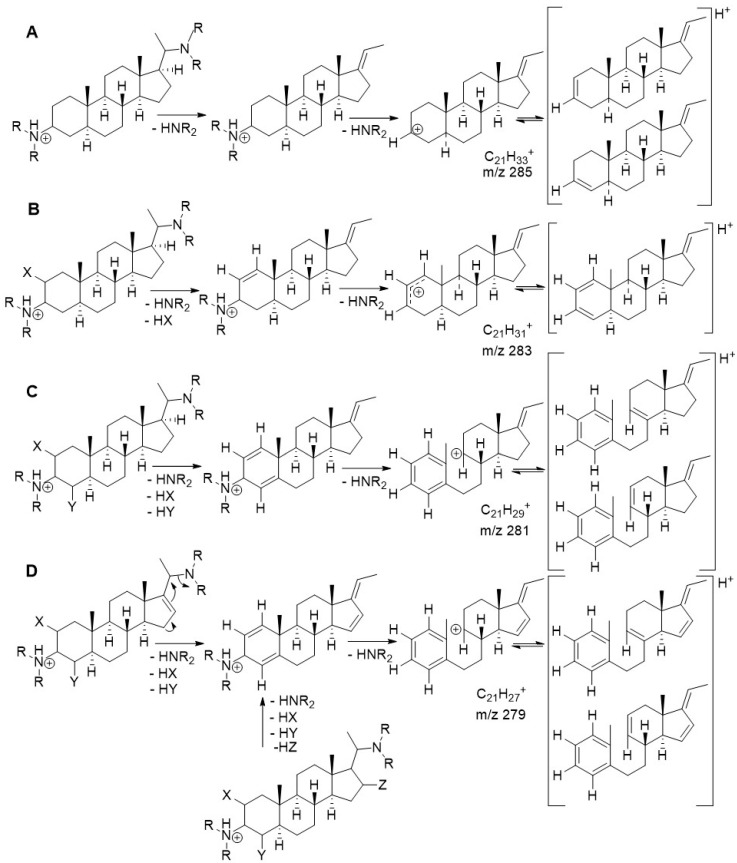
+ESI MS fragmentation pathways of amino-/amidosteroids depending on the substitution-/unsaturation pattern in the steroid core. (**A**) fully saturated and further unsubstituted C-3-/C-20 diamine or diamides: core fragment (CF) at *m/z* 285; (**B**) singly substituted or unsaturated: CF at *m/z* 283; (**C**) double substituted or unsaturated: CF at *m/z* 281; (**D**) double substituted with additional unsaturation or triple substituted: CF at *m/z* 279, according to Musharraf et al. [9] (modified). The previous authors formulated the system resulting from the loss of the C-20 substituent as a vinyl group (double bond from C-20 to C-21). In the course of the present study, quantum mechanical calculations were carried out which show that the ethylidene group formulated here is energetically much more favorable (see Section 2.4). Furthermore, bond breaking between C-4 and C-5 under formation of a double bond between C-3 and C-4 and localization of the positive charge at C-5 as well as breaking between C-9 and C-10 under formation of a C-10=C-1‒C-2=C-3 diene with the positive charge at C-9 were postulated for cases A and B upon elimination of the C-3-amino- or amido substituent [9]. In the course of this study, quantum mechanical calculations indicated that a bond breaking (between C-9 and C-10) is predicted by theory only in cases C and D, where this can lead to an aromatic system (see Section 2.4).

The fragmentation observed in the present study is in full agreement with the common characteristic ions described by Musharraf et al. [9]. These authors reported that such amino-/amidosteroids occurring in Buxaceae (*Pachysandra, Sarcococca*, etc.) show very informative fragmentation patterns in their +ESI mass spectra that allow clear conclusions on their substitution pattern [9]. In particular, diagnostic fragments of the main steroid core were found to occur, depending on the number of substitutions and double bonds in this core, which were used here to obtain plausible structural assignments. These fragments will henceforth be termed “core fragments” (CFs). CFs can occur at *m/z* 285, 283, 281 and 279, depending on the number of substituents/degree of unsaturation, typically in ring A of the steroid nucleus (see Figure 4). In addition to these previously reported fragmentation pathways, a number of further key fragments could be assigned in the present study, based on their exact masses and known general fragmentation rules (see spectra and fragmentation schemes in the Supplementary Material, Figures S1–S18).

Compound **4** (6.82:481; see Figure S4, Supplementary Material) differed from the known pachysandrine A (**6**, Figure S6) [13] by having a molecular mass that was lower by 42 units. From the [M + H]^+^ ion (C_31_H_49_N_2_O_2_^+^), it displayed a loss of the dimethylamino group (C_2_H_7_N, 45 Da) and H_2_O (18 Da), leading to the fragment at *m/z* 418 in analogy to **6**, which lost the amino group along with acetic acid (60 Da) leading to the same fragment. The mass spectrum of **4** was otherwise essentially identical to that of **6** with a CF at *m/z* 283 and the characteristic fragments of the *N-*methylated benzamide unit (*m/z* 136, base ion) and the benzoyl cation (*m/z* 105) so that **4** is very likely to represent the free alcohol derived from the acetate **6**—i.e., deacetylpachysandrin A.

Compounds **5**, **10** and **15** (Figures S5, S10 and S15, Supplementary Material, respectively) also displayed the mentioned typical fragments of an *N-*methyl benzamide moiety, most likely located at C-3 as in **6**. Furthermore, all three feature the CF at *m/z* 283 (C_21_H_31_^+^), indicating one further substituent in ring A besides the amide group at C-3. From its [M + H]^+^ ion (C_32_H_49_N_2_O_3_^+^) at *m/z* 509, compound **5** (7.15: 509) suffers a neutral loss of 73 Da (C_3_H_7_NO) which is likely to represent the substituent at C-20 and corresponds to an *N-*Methylacetamide moiety such as, e.g., present in known compounds such as Epipachysamine A [13] and Saracodine [14]. The fragment at *m/z* 436 (C_29_H_42_NO_2_^+^), resulting from this loss, further loses a molecule of water, leading to the fragment at *m/z* 418 (C_29_H_40_NO^+^); this loss indicates that the single additional substituent is an OH group, either at C-2 or at C-4, leading to the overall structure as depicted. The [M + H]^+^ ions of **10** and **15** (9.22:523, C_32_H_47_N_2_O_4_^+^ and 9.99:537, C_33_H_49_N_2_O_4_^+^, respectively) are characterized by a common loss of acetic acid (60 Da) from an ester group as in **6**. This is followed by losses of 45 and 59 Da, respectively, both leading to a fragment at *m/z* 418 as above. Interestingly, the former loss, in **10**, does not correspond to a dimethylamino moiety as in compounds **4** and **6,** but rather represents CH_3_NO (i.e., NH_2_CHO), as determined by the exact mass. This neutral loss is expected in the case of a formamido substituent at C-20. Likewise, the loss of 59 Da in **15** corresponds to C_2_H_5_NO (i.e., NH(CH_3_)CHO)—i.e., an *N-*methylformamido group. Interestingly, the whole sidechain from C-17, i.e., the *N-*methylformamide group along with C-20 and C-21, was observed as a relatively intense fragment at *m/z* 86 (C_4_H_8_NO^+^), which also occurs in further compounds with a C-20-*N-*methylformamide group (**11**–**17**, see below). Aminosteroids with both nonmethylated and *N-*methylated formamide moieties, such as, e.g., iso-*N-*formylchonemorphine and *N-*(formyl(methyl)amino)salonine-B [15], are known from Buxaceae. Thus, the structures of **10** and **15** are very likely, as depicted.

Compounds **12** and **14** (9.79:441and 9.88:515, [M + H]^+^: C_28_H_45_N_2_O_2_ and C_31_H_51_N_2_O_4_, respectively; Figures S12 and S14, Supplementary Material) share the CF at *m/z* 283 with those just described. Instead of the fragments due to the *N-*methyl benzamide, they display signals corresponding to tigloyl-, angeloyl- or senecioylamide (tig/ang/sen) groups, which are also commonly observed in this class of alkaloids [9]. In the case of **12**, fragments at *m/z* 100 (C_5_H_8_NO^+^) and 83 (C_5_H_7_O^+^) are observed, and in agreement with neutral losses of 99 and 82 Da, which indicate an unsubstituted tig/ang/sen amide moiety, as typically located at C-3 of such amidosteroids. In the case of **14**, the corresponding fragments appear at *m/z* 114 (C_6_H_10_NO^+^) and 83. The former indicates an *N-*methylated tig/ang/sen amide group. Akin to **15**, **12** and **14** suffer a neutral loss of 59 Da corresponding to C_2_H_5_NO and also show the fragment at *m/z* 86—i.e., they should both have an *N-*methylformamido group at C-20. Compound **14**, akin to **6**, **10** and **15,** also shows the loss of 60 Da (acetic acid) and should thus, as the mentioned compounds do, possess an acetoxy group, most likely in ring A. This is not the case in compound **12**, whose elemental formula does not allow for another substituent besides the amide groups at C-3 and C-20, but instead requires a further degree of unsaturation. In this case, the occurrence of the CF at *m/z* 283 must indicate the presence of a double bond within the steroid core, which could be located between C-16 and C-17, as observed in various Buxaceae alkaloids [16,17,18]. This would be supported by the rather high abundance of *m/z* 283 (>50%) in **12** since, in this case, the additional double bond resulting from the loss of the *N-*methyl formamide group from C-20 would lead to a conjugated diene system (C-20=C-17-C-16=C-15) which could energetically favor the formation of this ion. This is also supported by the high abundance of the ion at *m/z* 86 representing the complete sidechain from C-17, which in the case of **12** is the base ion, which should be due to the more favorable cleavage at this side due to the 16-17 double bond. The structures of **12** and **14** are therefore likely, as depicted.

Another compound with a (nonmethylated) tig/ang/sen group at C-3 and an *N-*methyl formamide group at C-20 is compound **16** (10.10:443; [M + H]^+^: C_28_H_47_N_2_O_2_^+^; Figure S16, Supplementary Material). Apart from these two substituents, no further substitution or unsaturation in the steroid core is possible, which is confirmed by the occurrence of the CF at *m/z* 285 (C_21_H_33_^+^; compare Figure 4A). The likely structure is as depicted.

Compound **18** also shows the typical fragments and neutral losses for a tig/ang/sen amide (Figure S18, Supplementary Material). Its CF at *m/z* 281 (C_21_H_29_^+^) indicates the presence of two further substituents or double bonds in the steroid core. From the fragment resulting from the [M + H]^+^ at *m/z* 412 (C_27_H_42_NO_2_^+^) by neutral loss of the tig/ang/sen-amide group (−99 Da, *m/z* 313, C_22_H_33_O^+^), a further neutral loss of 32 Da (CH_4_O) was observed, which corresponds to a loss of methanol leading to the CF. It can therefore be deduced that the molecular structure contains a methoxy substituent. Since the elemental formula does not allow for a further substituent but requires two further degrees of unsaturation, there should be an ethylidene group at C-17, as in **1** and **3**, along with a further double bond in the steroid core. The formation of the base fragment at *m/z* 157 and an intense related fragment at *m/z* 131 can only be rationalized with the methoxy substituent at C-16. The position of the double bond was assigned as in the known 16-methoxy-substituted Sarcococca-alkaloid sarcosalgmin [19], leading to the depicted structure of compound **18**.

The [M + H]^+^ ion of compound **9** (8.02:455; Figure S9, Supplementary Material) had the elemental formula C_29_H_47_N_2_O_2_^+^ and the CF was observed at *m/z* 279 (C_21_H_27_) in this case, pointing to the presence of a double bond and two further substituents (or a keto group) in the structure. The quasimolecular ion at *m/z* 455 suffers a neutral loss of 31 Da (CH_5_N corresponding to methyl amine), which indicates the presence of a methyl amino group, possibly at C-20. From the resulting fragment at *m/z* 424 (C_28_H_42_NO_2_), water is eliminated (18 Da) to yield C_28_H_40_NO^+^ at *m/z* 406. The most intense fragment is observed at *m/z* 314 (C_21_H_32_NO^+^) so that a loss of C_7_H_10_O (110 Da) from that at *m/z* 424 can be postulated. This loss is in agreement with the presence of a prominent fragment at *m/z* 111 (C_7_H_11_O^+^). A further prominent fragment, only a little less intense than the base peak, occurs at *m/z* 122 and has the formula C_7_H_8_NO, which corresponds to a protonated benzamide such as found in compound **8**, the known Epipachysamine D. Notably, however, the corresponding fragment seemingly indicating an *N-*methyl benzamide (C_8_H_10_NO^+^ at *m/z* 136, cf. compounds **4**, **5**, **6**, **10**, **15**) was also observed. The simultaneous presence of an *N-*methylated and unmethylated benzamide moiety as well as a cleavable moiety C_7_H_11_O is clearly not accommodated by the overall elemental formula, C_29_H_46_N_2_O_2_. The overall number of degrees of unsaturation is six, of which four are at least required for the tetracyclic steroid skeleton. Thus, the presence of an aromatic side chain is not possible so that the two putative “benzamide-like” fragments must in this case have a different origin. Indeed, their formation can be rationalized by cleavage of ring B of the base ion (*m/z* 314) by two slightly different pathways as shown in Figure S9, Supplementary Material). The above-mentioned neutral loss of 92 Da and the fragment at *m/z* 111, on the other hand, clearly point to a cleavable side chain of the formula C_7_H_11_O, which would correspond to a cyclohexane carboxamide or a singly unsaturated n-heptane carboxamide group. The presence of mass signals at *m/z* 218 and 204, though small, according to literature indicates the presence of a steroid with a double bond between C-2 and C-3 and an oxo group at C-4 such as sarcovagine D [9]. This known compound, akin to compound **9**, also yielded a fragment at *m/z* 314 and features the CF at *m/z* 279. Thus, assuming the same basic skeleton as in sarcovagine D, but with a monomethylamino group at C-20 and a C-3 amide moiety with an acyl fragment of elemental structure C_6_H_11_CO^+^ results in the depicted strucure of compound **9**. The very low abundance of the fragments representing the whole amide (i.e., C_6_H_11_CONH_3_^+^ at *m/z* 128) and the corresponding neutral loss of 127 Da (at *m/z* 328 and 297) could also be explained by the fact that the amide group is located at an olefinic carbon where the elimination of the whole amide including the nitrogen atom is quite unfavourable. Instead, the fragment containing both the amino/ammonium group and the keto/enol groups (*m/z* 314) is the most stable ion in the spectrum, probably because these groups can directly interact with each other which will stabilize this species. Its tendency to lose either water (18 Da) or ammonia (17 Da) leading to the small fragments at *m/z* 296 and 297, or both, then yielding the low-abundant CF at *m/z* 279 is hence rather small. This taken together, the proposed structure of compound **9** is in full agreement with all major spectral features.

In the case of compound **7** (7.57:449; see Figure S7, Supplementary Material), similar to several others described here, the [M + H]^+^ representing C_30_H_45_N_2_O^+^ first undergoes a neutral loss of 45 Da (C_2_H_7_N) showing the presence of a dimethylamino group. The base ion at *m/z* 122 (C_7_H_8_NO^+^) indicates the presence of a simple benzamide moiety such as in compound **8**. Overall, the spectrum of **7** is very similar to that of the known **8** ([M + H]^+^ at C_30_H_47_N_2_O^+^, see Figure S8, Supplementary Material), from which it differs only by an additional degree of unsaturation, which –in view of the lack of any possibility for further substituents, should be due to a double bond in the tetracyclic steroid core. This is corroborated by the CF at *m/z* 283 (C_21_H_31_^+^; compare Figure 4). The benzoyl cation (C_7_H_5_O^+^, *m/z* 105) is formed in addition to the protonated benzamide ions in all compounds with a benzamide moiety. From the rather low intensity of this ion at *m/z* 105 in compounds **7** and **8** as compared with those in the *N-*methylbenzamide derivatives, it may be deduced that the formation of this ion in these cases is far less favourable than the formation of the ion representing the whole benzamide group. The position of the double bond in **7** was assigned by analogy to compound **3** (see above) and by the pattern of lower mass fragments (see fragmentation scheme in Figure S7), which could be rationalized, with difficulty, only if the double bond were in another position than between C-5 and C-6. A large variety of Pachysandra steroids with double bonds in this position have been described (e.g., [14,15,20,21]. Thus, overall, this results in the depicted structure of compound **7**.

The mass spectra of compounds **11**, **13** and **17** (Figures S11, S13 and S17, Supplementary Material) could not readily be translated into structural hypotheses by these known fragmentation pathways since their CFs could not be unequivocally determined. In all three cases, however, it appears clear from their elemental formulas ([M + H]^+^ ions representing C_29_H_45_N_2_O_3_^+^, C_29_H_47_N_2_O_3_^+^, and C_31_H_45_N_2_O_2_^+^, respectively) and overall fragmentation, that they are aminosteroids. All three contain an *N-*methylformamide moiety as becomes obvious from the characteristic neutral losses of 59 Da (C_2_H_5_NO) and by the prominent fragments at *m/z* 86 (see compound **15** above). Compounds **11** and **13** share most fragments and differ only in the nature of an acyl side chain obviously forming an ester in these cases. In the case of **11** (neutal losses 82 and 100 Da (C_4_H_6_CO and C_4_H_7_COOH), this is a tig/ang/sen ester, whereas in the case of **13**, a saturated C_5_-caboxylic acid ester, probably 2-methylbutyrate or isovalerate (neutal losses 84 and 102 Da, C_4_H_8_CO and C_4_H_9_COOH, respectively), must be present.

In the case of compound **2** (mass spectrum see Figure S2, Supplementary Material), no reasonable elemental formula could be obtained from the (+)ESI QTOF MS. The structure of these compounds must therefore remain uncharacterized.

### 2.4. Quantum Mechanical Assessment of the (+)ESI MS Fragmentation

Considering the structure of the known aminosteroids, **1** and **3,** with their ethylidene groups on the one hand, and their structures, as postulated by previous authors, as the fragments resulting from neutral loss of the C-20 substituent with a vinyl group on the other [9], the question arose whether these fragments might not possess an ethylidene group at C-17 rather than a vinyl group. Therefore, quantum mechanical calculations were carried out in order to assess the stability of either of the possible elimination products. The calculations were carried out using density functional theory (DFT) at the B3LYP\6-31G+(d,p) level and performed on part of the steroid nucleus comprising rings C and D along with the C_2_-side chain in the various possible forms after elimination of the substituent (see Table 3). For the simple case with a fully saturated cyclopentane ring D (as observed in most of the compounds of this study), the results clearly show that the ethylidene form with an E-configured double bond between C-17 and C-20 (case A3 in Table 3) is far more stable than either the *Z-*form or the vinyl form with the double bond between C-20 and C-21 (cases A2 and A1 in Table 3, respectively). Similarly, in the case of compounds with a double bond in the D-ring between C-16 and C-17 (represented in this study by compound **12**), the results of the calculations show that formation of a Δ^17,20^ *Z-*configured C-15=C-16-C-17=C-20 conjugated diene (case B3 in Table 3) is far more feasible than either the corresponding E-form or the isomeric C-16=C-17-C-20=C-21 diene (cases B2 and B1 in Table 3).

Thus, in both cases the resulting fragments appear to have ethylidene-type structures rather than a vinyl group attached to C-17.

**Figure 5:**
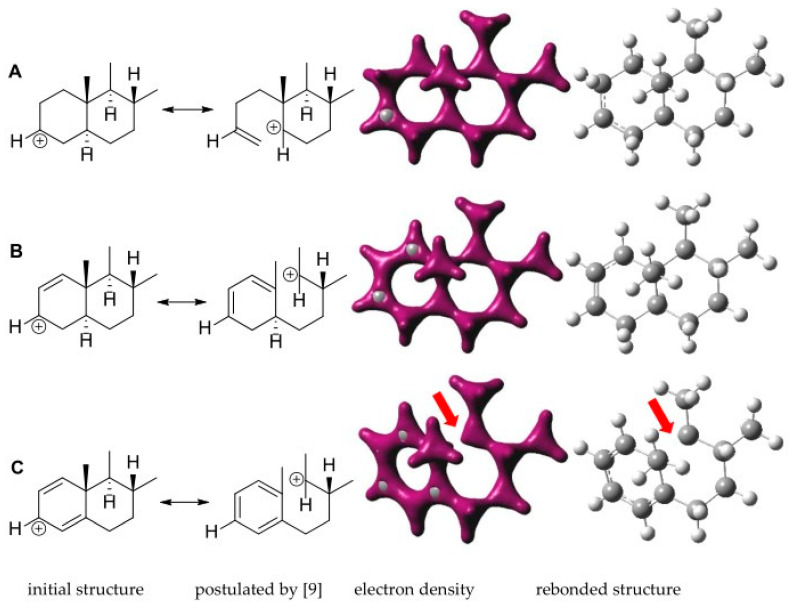
Results of DFT energy minimization of the cations resulting from loss of the C-3 amino substituent. Model fragments depicted as initial structures (but without localization of the positive charge) were minimized at the B3LYP\6-31G+ (d,p) level. The structures in (**A**–**C**) correspond to cases A, B and C in Figure 4. The electron density maps as well as a structure with bond orders redrawn after minimization are shown to illustrate the localization of (partial) bonds. Note that C is the only case in which bond breaking is predicted by the theoretical models (red arrow). The rebonded structures correspond to those shown for the various CFs in Figure 4.

Another question that arises from inspecting the structures in Figure 4 regards whether the cations resulting from the loss of the C-3 amino group in cases A through D are likely to show breaking of bonds in the positions of the steroid ring system postulated by previous authors [9]. To investigate this, the respective cations of model fragments representing these structures were energy minimized using the same level of theory as applied in the above-mentioned calculations. The resulting structures with their electron density maps and bond orders are shown in Figure 5.

From these theoretical considerations, it may be expected that the postulated [9] bond breaks in the CFs of compounds considered here will only occur in cases where the steroid core has two substituents in ring A which can be eliminated under the formation of double bonds, as in case C, which can then be stabilized to a quasi-aromatic system—i.e., in the CFs of mass 279 and mass 281 (compare Figure 4).

### 2.5. Distribution of Presumably Active Compounds in the Various Fractions of PS_AEF

**Figure 6:**
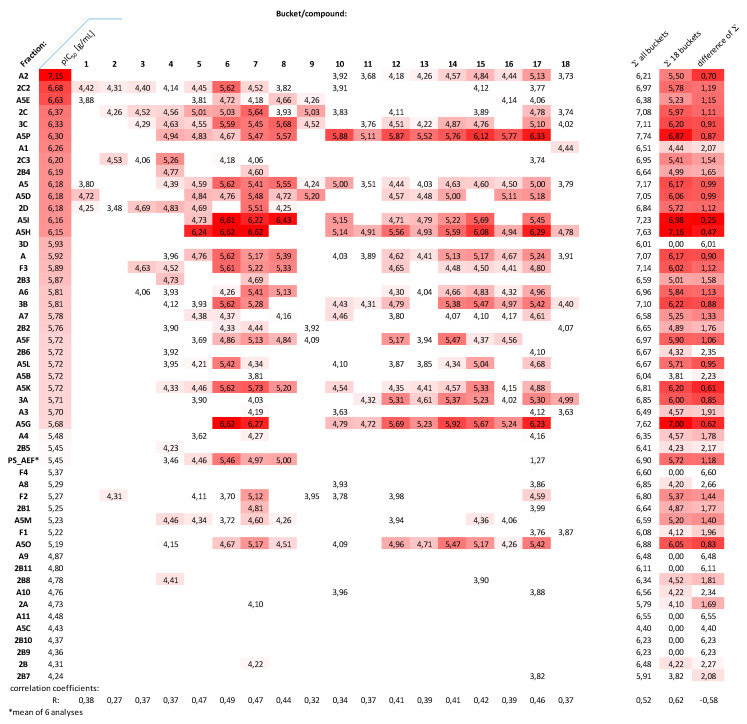
Heat map representation of the distribution and amount (log values of bucket intensity values) of the 18 selected compounds in the various fractions in relation to their anti-Tbr activity (−log IC_50_ (g/mL) = pIC_50_), with a more intense red indicating higher activity and higher intensity. Correlation coefficients with the pIC_50_ values are shown for each individual compound, for the sum of logarithmic total intensity of all buckets, for the sum of the 18 selected compounds/buckets’ intensity data, as well as for the difference between these two sums representing the log value of the fraction of the 18 compounds to the total of each fraction.

**Figure 7:**
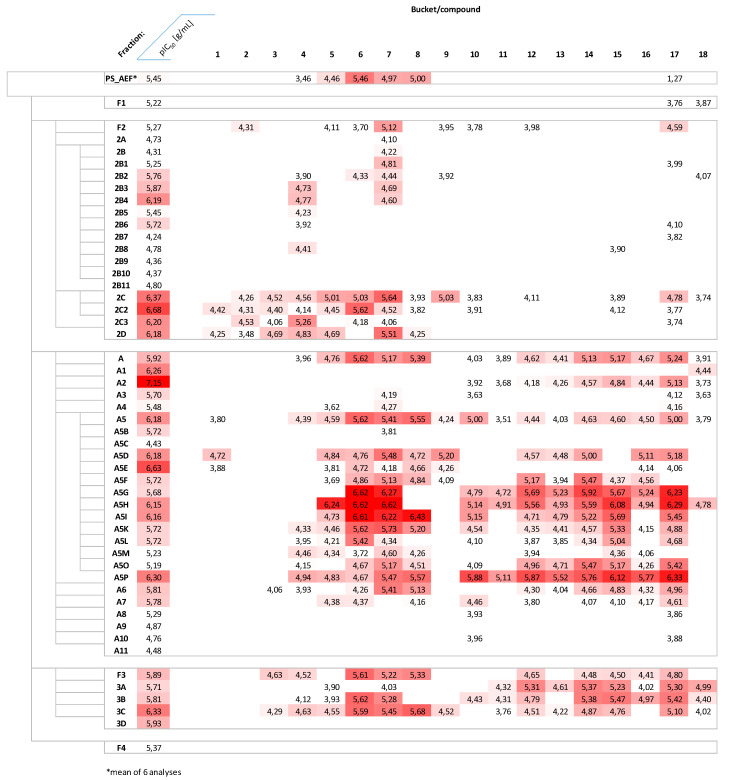
Heat map representation of the distribution and amount (log values of bucket intensity values) of the 18 selected compounds in the various fractions in relation to their anti-Tbr activity (−log IC_50_ (g/mL) = pIC_50_), with a more intense red indicating higher activity and higher intensity. Fractions are ordered according to their occurrence in the fractionation scheme (compare Figure 8, Material and Methods section).

With the exception of compounds **1**, **3**, **6** and **8**, none of the steroids described in this study has previously been reported as a constituent of P. terminalis, to the best of our knowledge. In order to obtain further information on the possible contributions of these 18 compounds highlighted by the PLS model, their intensity values (logarithmized bucket values) in each of the tested fractions as well as the overall PS_AEF were extracted from the total bucket table. They are shown, along with their activity data (negative logarithms of the IC_50_ data (in g/mL), i.e., pIC_50_ values, against Tbr) in the form of color-coded heatmaps in Figure 6 and Figure 7.

In Figure 6, the fractions are arranged in descending order of their antitrypanosomal activity. It can be seen that there is an overall trend of the more active fractions to contain higher amounts of the 18 compounds. Fractions with low activities (below that of the total PS_AEF) contain only few or none of these compounds, whereas higher intensities and numbers of these compounds appear to be present in the more active fractions. The sum of the 18 compounds’ bucket values (a) thus shows a significant degree of correlation (R = 0.62) with the pIC_50_ values. Since the amounts of the compounds in each fraction and their relative contribution to the overall composition is not known, this sum (Σa) was related to the total sum of all logarithmic bucket values (Σb). The difference (Σb-Σa, representing the ratio of the original intensity values in nonlogartihmic form) did not show a higher, but rather somewhat lower, correlation coefficient with the activity data. The intensity values of each compound were also investigated individually for correlations with activity. However, none of them reached an R value > 0.5, which indicates that none of the single compounds on their own is significantly more influential on activity than the others. This result rather supports the idea of a complex activity profile due to the influence of various compounds. It should not remain unmentioned that some of the more active fractions did not show particularly high values for any of the highlighted compounds, so a question arises as to whether all active principles were covered by the analyses performed here. However, the trend observed with an overall increase in the 18 compounds’ contents with activity makes it quite plausible that these compounds are responsible, at least to a significant extent, for the antitrypanosomal activity observed.

To investigate the distribution of the presumably active compounds over the fractionation scheme, these data were also ordered according to the sequence of fractionation steps (Figure 7). It becomes clear that the overall content of the compounds of interest appears to be low in the early fractions (F1—none detected, F2 and its early subfractions) and increases in the later, more active fractions (late subfractions of F2, fractions A and F3), while they were, again, absent in the last fraction, F4. It also appears that there are two main clusters of such compounds in the earlier (compounds **1**–**9**) and later (compounds **10**–**18**) retention time regions, of which the latter only appears in more significant amounts in the subfractions of A and F3 while the former already is found at rather significant levels in the later subfractions of F2. Since the separation protocol is based mainly on normal phase CC separation, this indicates that the more active compounds occur within a medium polarity window—i.e., very nonpolar (early fractions) and nonpolar (late fractions) compounds do not contribute to the overall activity. Further studies with the aim of targeted isolation of the compounds described here from the very complex mixture will have to investigate in detail the significance of these single constituents, and, possibly, combinations thereof, for the antitrypanosomal effect of *P. terminalis* and its PS_AEF.

## 3. Materials and Methods

### 3.1. Plant Material

Aerial parts of *Pachysandra terminalis* Sieb. and Zucc. were harvested from the same population in the Medicinal Plant Garden of the Institute of Pharmaceutical Biology and Phytochemistry (IPBP) of the University of Münster in October 2015 (small scale extraction) and in July–October 2017 (large scale extraction). The plants were dried at 40 °C and then stored in paper bags until further use. A voucher specimen (voucher number 523) was deposited at the IPBP herbarium.

### 3.2. General Methods

Column chromatography (CC) was performed at ambient pressure and room temperature using silica gel 60 as the stationary phase in glass columns. The columns of appropriate size (i.e., large enough to hold about 100-fold mass of silica in relation to the sample to be separated) were filled with a slurry of the stationary phase in the starting eluent, left for 3 h and then conditioned at a low flow rate. The silica bed was then covered with a thin layer of sea sand and the samples were usually introduced in dissolved form; in some cases, poorly soluble samples were suspended in DCM and left to dry with a small amount of silica gel which was then strewn on the bed of stationary phase.

Analytical thin layer chromatography (TLC) was performed on silica 60 F 254 gel-coated aluminum sheets (20 × 20 cm; Merck, Darmstadt, Germany), usually developed over a distance of 8 cm and then observed under UV light (254 nm) followed by spraying with Dragendorff reagent (composition). For analytical purposes (establishment of appropriate eluent systems for CC, fraction monitoring), the sheets were cut in two 10 cm halves. The samples were applied in bands (width 1 cm) and the plates developed over a distance of 8 cm with the eluent mixtures mentioned below. For preparative TLC (pTLC), the samples were applied as bands of 18 cm width and the plates developed over a distance of 18 cm. The bands were then detected under UV 254 nm and/or by spraying narrow strips with Dragendorff reagent, and were marked with a pencil and had silica scraped off using a steel spatula. The analytes were then extracted from the silica powder with an appropriate solvent.

### 3.3. Extraction and Fractionation

The protocol applied for extraction and obtention of the alkaloid-enriched phase was similar to that applied in our studies on *Buxus sempervirens* [8,22].

For the initial biological tests, 27 g of dried and ground plant material was extracted exhaustively with dichloromethane (DCM) and then partitioned by acid/base extraction as detailed below.

For the following detailed investigation, 2.6 kg of dried and ground plant material was extracted with DCM in a Soxhlet apparatus until the supernatant was clear and colorless. The extract was evaporated to dryness on a rotary evaporated at max. 40 °C to yield 249 g (9.6%) of crude extract.

The alkaloid-enriched fraction (PS_AEF) was obtained by dissolving portions of 10 g of the crude extract in 40 mL of DCM. The solutions were extracted five times with 70 mL 0.1 M aqueous hydrochloric acid in a separatory funnel. The lipophilic DCM phases (PS_NF) were evaporated to dryness yielding a residue of 1.3 g. The acid phases were combined and alkalinized with aqueous 2 M sodium hydroxide solution. The combined alkaline phases were then extracted 5 times with the 1.5-fold volume of DCM. The resulting phases (aqueous residual phase PS_WAT and alkaloid-enriched organic phase (PS_AEF) were evaporated to dryness by lyophilization and rotary evaporation, respectively. The resulting dry fractions amounted to 18 g (0.7% of plant material dry weight) PS_AEF, along with 2.8 g PS_WAT.

**Figure 8:**
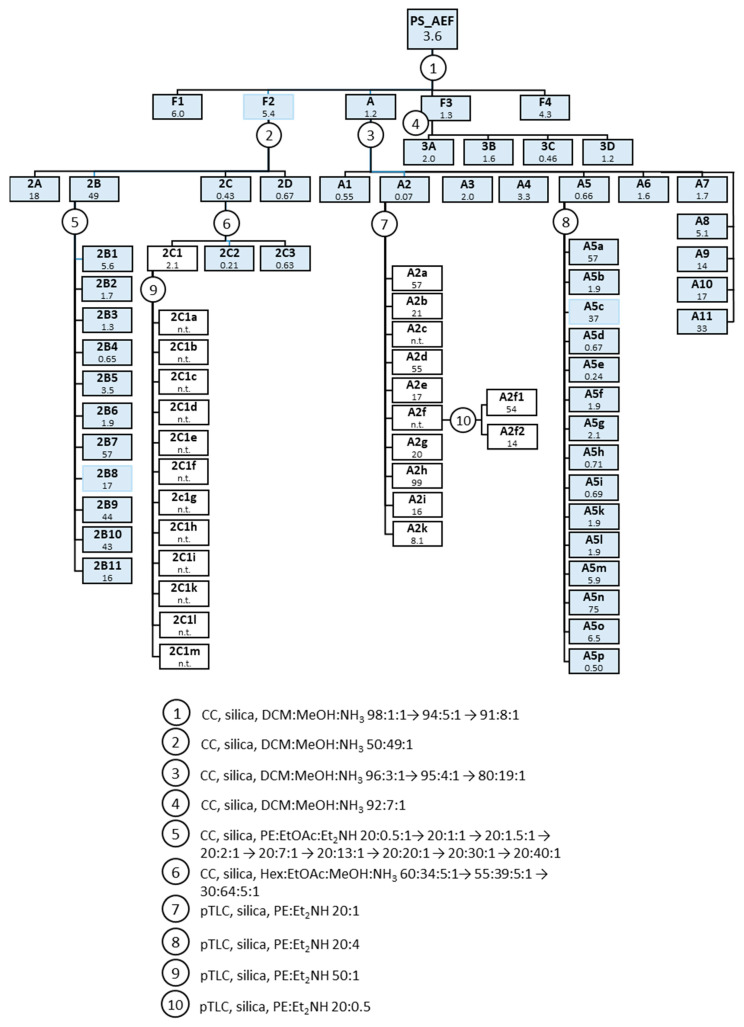
Fractionation of PS_AEF; IC_50_ values (µg/mL) of individual fractions against Tbr are reported below the fraction denominations. Fractions in light blue boxes were included in the PLS modeling. Three outliers not included in the final PLS model are marked by light blue frames.

A total of 18 g PS_AEF was separated by column chromatography (CC) on 1824 g silica using a solvent gradient of DCM/methanol (MeOH)/ammonia (NH_3_) of increasing polarity—i.e., increasing MeOH concentration as follows: DCM:MeOH:NH_3_ 98:1:1 (2.2 L), 94:5:1 (6.8 L), 91:8:1 (2.7 L). After TLC control (silica F254, dragendorff reagent), the eluates were combined in five fractions: F1 (1.7 g), F2 (1.8 g), A (1.3 g), F3 (3.8 g) and F4 (0.5 g). The fractions were tested for activity against *Tbr* and yielded IC_50_ values of 6.0, 5.4, 1.2, 1.3 and 4.3 µg/mL, respectively. They were then subfractionated using various CC and preparative thin layer chromatography (pTLC) methods as summarized in Figure 8.

### 3.4. UHPLC-(+)ESI QTOF MS and MS/MS Analysis

All analyses were performed on a Bruker Daltonics (Bremen, Germany) micrOTOF QII quadrupole/time-of-flight mass spectrometer with an Apollo electrospray ion source operated in positive ionization mode and coupled to a Dionex Ultimate 3000 RS Liquid Chromatography System (Idstein, Germany) with a Dionex Acclaim RSLC 120, C18 column (2.1 × 100 mm, 2.2 µm, protected by an EXP guard column 3 × 5 mm, C-18, 1.8 µm) using a binary gradient of water and acetonitrile, both with 0.1% formic acid. The LC system was additionally equipped with a Dionex Ultimate DAD-3000 RS (wavelength range of 200–400 nm) UV/Vis detector.

The samples were dissolved in methanol at a concentration of 1 mg/mL. The injection volume was 2 µL. Separations were performed using a binary gradient of A: water, B: acetonitrile, both with 0.1% formic acid at a flow rate of 0.4 mL/min: 5% B (0–0.4 min); 5–100% B (0.4–9.9 min); 100% B (9.9–15 min); re-equilibration at 5% B (15.1–20.0 min). The column was themostatted to 40°C. Detection: DAD, 200–400 nm; (+)ESI QTOF MS was performed in auto MSMS (MS^2^) mode including the three most abundant ions between *m/z* 200 and 1500 in every forth (full scan) spectrum. Acquisition rate: 5 Hz, mass range: *m/z* 50–1500; nebulizer gas N_2_, 5 bar; dry gas N_2_, 9 L/min, 220 °C; capillary voltage 4500 V; end plate offset −500 V; transfer time 70 µs; collision gas N_2_; the collision energy for MS^2^ spectra was 40 eV and the isolation width 5 *m/z* units. Internal dataset calibration (HPC mode) was performed for each analysis using the mass spectrum of a 10 mM solution of sodium formate in 50% aqueous isopropanol that was infused during LC re-equilibration using a divert valve equipped with a 20 µL sample loop.

### 3.5. Data Pretreatment of LC–MS-Data

The raw data from 58 analyses (52 fractions and subfractions with determined IC_50_ values and sufficient amount for analysis + 6 analyses of the crude alkaloid-enriched fraction PS_AEF, all performed in one coherent series) were converted into a bucket table using the software Profile Analysis v. 2.1 (Bruker Daltonics, Bremen, Germany). To this end, all data in the retention time (tR) range of1–15 min and in the mass range of *m/z* 90–1450 were submitted to the advanced bucketing function using a time window of 0.2 min and a mass window of 50 mDa; only buckets occurring in 2 or more samples were kept. The signal to noise threshold was set to 10, the correlation coefficient threshold to 0.7, the minimum compound length to 3 spectra and a smoothing width of 1 was applied. The raw intensity data were not normalized and transformed to logarithmic scale. This resulted in a total of 1238 bucket variables of the form <tR(min):*m/z*>.

### 3.6. Multivariate Data Analysis—PLS Regression Modeling

The bucket table (1238 bucket variables × 58 analyses) was exported and analyzed with SIMCA (v. 16.0.1, Sartorius stedim biotech, Umeå, Sweden). The activity data against *Tbr* (negative decadic logarithms of IC_50_ (g/mL) = pIC_50_ values of each sample were added to the table as the dependent (Y-) variable. All variables were scaled to unit variance before the PLS regression modelling. The models were cross-validated applying the leave-one-out validation scheme. In an initial PLS model including all 58 samples, three samples, 2B8, A5C and F2, showed very high residuals, leading to poor performance in cross-validation (Q^2^ = 0.459). These three samples were therefore omitted so that a model with good statistical performance was obtained with the remaining 55 analyses. The statistical performances of the resulting PLS models (coefficients of determination of the model calibration, R^2^ and of the cross-validation predictions, Q^2^) with 1–5 latent variables (PLS components) are summarized in Table 4. From the significant increase in the cross-validated coefficient of determination Q^2^ between models with 3 and 4 components and the fact that there was no further increase in Q^2^ with a 5th component, the optimum number of components was found to be 4. This 4-component model was then used to select buckets/compounds with presumably strong influence on the bioactivity of PS_AEF.

### 3.7. Biological Testing

In vitro assays for the bioactivity of crude extracts and fractions against *Trypanosoma brucei rhodesiense* (*Tbr*, bloodstream trypomastigotes, STIB 900 strain), Trypanosoma cruzi (*Tc*, intracellular amastigotes, Tulahuen C4 strain, in L6 rat cells (see below)), *Leishmania donovani* (*Ld*, axenic amastigotes, MHOM-ET-67/L82 strain), *Plasmodium falciparum* (*Pf*, intra-erythrocytic forms, NF54 strain), and cytotoxicity test against mammalian cells (L6-cell-line from rat-skeletal myoblasts) were performed at the Swiss Tropical and Public Health Institute (Swiss TPH, Basel, Switzerland), according to established protocols and as previously described [23] (for a brief repetition of these methods, see Supplementary Materials).

### 3.8. Quantum Mechanical Calculations

3D molecular structures of the model fragments were generated with MOE v.2018.1 (Chemical Computing Group, Montreal, Canada). Their geometry was optimized by performing a low mode dynamics (LMD) conformational search using the AMBER EHT force field in each case. The resulting geometries were energy minimized using the semiempirical PM3 hamiltonian, then exported in *.mol2 format and submitted to a density functional theory (DFT) energy minimization within the B3LYP functional with the 6-31G+ (d,p) basis set in Gaussian 03W (Gaussian Inc., Wallingford, CT, USA). Electron density maps and rebounded structures were generated using GaussView 3.0 (Gaussian Inc.).

## 4. Conclusions

The alkaloid fraction from the DCM extract of *P. terminalis* aerial parts was found to be a potent trypanocide with high activity and selectivity against *T. brucei rhodesiense*. Due to the complexity of the steroid alkaloid mixture in the numerous fractions obtained and tested during the activity-guided fractionation, multivariate statistics were applied. Eighteen compounds were highlighted as the result of partial least squares regression modelling, which are likely to be of importance for the high overall activity. Thorough analysis of the mass spectra (MS and MS^2^) of these compounds allowed dereplication of four steroid alkaloids (**1**, **3**, **6** and **8**) previously known as constituents of the title plant as well as establishment of structural details in ten other cases (compounds **4**, **5**, **7**, **9**, **10**, **12**, **14**–**16**, **18**), not previously reported as constituents of *P. terminalis*. Thorough analyses of the MS^2^ fragmentation, along with quantum mechanical calculations on certain fragmentation details were performed, which yielded further insights into the mass spectral behavior of this class of natural products. Further studies will investigate the contribution of these promising single constituents alone or in combination with the antitrypanosomal activity of *P. terminalis*.

## Figures and Tables

**Figure 3:**
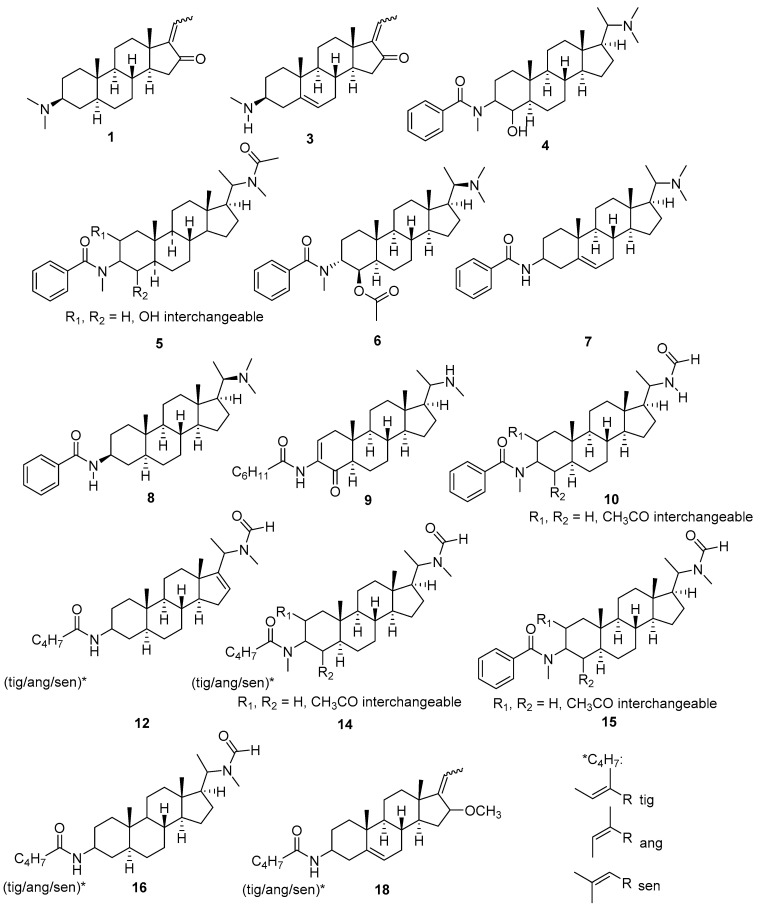
Structures of the 18 compounds selected from the PLS model. Please note that compounds **1**, **3**, **6** and **8** were dereplicated by comparison with known compounds from *P. terminalis* whereas the other structures were deduced from the mass spectra in agreement with previous data on related compounds ([9], compare Figure 4 and Figures S1–S18, Supplementary Material). Stereochemical assignments were kept in the case of known compounds but omitted except for the steroid core in the case of tentatively assigned compounds.

**Table 1:** Biological activity of *P. terminalis* crude dichloromethane (DCM) extract and its fractions. Data are expressed as IC_50_ values in µg/mL.

Sample	*Tbr*	*Tc*	*Ld*	*Pf*	Cytotox (L6)
PS_DCM	4.2 ^1^	93 ^1^	41 ^1^	>50 ^1^	n.t.
PS_AEF	0.39 ± 0.08 ^2^	8.5 ^1^	34 ^1^	1.8 ^1^	9.5 ± 2.8 ^2^
PS_NF	7.0 ^1^	60 ^1^	>100 ^1^	28 ^1^	>100 ^1^
Pos. control ^2^	0.004 ± 0.001 ^3^	0.57 ± 0.04 ^4^	0.09 ± 0.03 ^5^	0.002 ± 0.001 ^6^	0.004 ± 0.001 ^7^

^1^ Determined only once. ^2^ Mean of two independent determinations ± deviation from the mean. ^3^ Melarsoprol, ^4^ benznidazole, ^5^ miltefosine, ^6^ chloroquine and ^7^ podophyllotoxin.

**Table 3:**
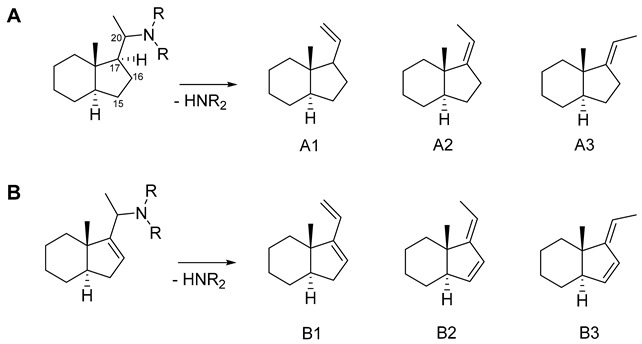
Results of quantum mechanical energy calculations (density functional theory (DFT) at the B3LYP\6-31G+(d,p) level) for the possible fragments arising from loss of the C-20 substituents. A: Saturated ring D; B: Ring D with double bond between C-15 and C-16. Calculations were carried out with the perhydro indane model compounds as depicted.

	ΔE ^1^ (kcal/mol)	% ^2^
Product	B3LYP\ 6-31G+ (d,p)	
A1 ^3^	4.76	0.03
A2	3.64	0.22
A3	0.00	99.8
B1	1.23	10.7
B2	1.81	4.0
B3	0.00	85.3

^1^ Energy difference from minimum. ^2^ Percentage in a hypothetical ensemble of the three possible forms obtained from the Boltzmann distribution. ^3^ Lower one of two possible rotamers.

**Table 4:** Statistical characteristics of the PLS model used to select presumably active constituents.

N Components	R^2^	Q^2^ *
1	0.442	0.137
2	0.688	0.166
3	0.877	0.473
**4**	**0.949**	**0.662**
5	0.982	0.628

* Leave-one-out cross-validation.

## Data Availability

The data presented in this study are available on request from the corresponding author. The data are not publicly available due to the requirements of ongoing research.

## Supplementary Materials

The following are available online, Description of bioassay methods; +ESI QTOF mass spectra of compounds **1**–**18** with fragmentation pathways (Figures S1–S18).

## Sample Availability

Samples of the compounds are not available from the authors.
