# Supplementary material for: The Alkaloid-Enriched Fraction of Pachysandra terminalis (Buxaceae) Shows Prominent Activity against Trypanosoma brucei rhodesiense
Source: Molecules. 2021 Jan 23;26(3):591. doi: 10.3390/molecules26030591 (PMC7865568; doi:10.3390/molecules26030591)
Supplement: Supplementary file 1 [file molecules-26-00591-s001.pdf]

Supplementary Material

The Alkaloid Fraction of *Pachysandra terminalis* (Buxaceae) shows prominent Activity against *Trypanosoma brucei rhodesiense*.

Dagmar Flittner[1], Marcel Kaiser[2,3], Pascal Mäser[2,3], Norberto P. Lopes[4] and Thomas J. Schmidt[1,*]

[1] Institute of Pharmaceutical Biology and Phytochemistry (IPBP), University of Münster, PharmaCampus, Corrensstr. 48, D-48149 Münster, Germany; dagmar.flittner@web.de (D.F.); thomschm@uni-muenster.de (T.J.S.);

[2] Swiss Tropical and Public Health Institute (Swiss TPH), Socinstrasse 57, CH-4051 Basel, Switzerland; marcel.kaiser@unibas.ch (M.K.); pascal.maeser@swisstph.ch (P.M.)

[3] University of Basel, Petersplatz 1, CH-4003 Basel, Switzerland

[4] NPPNS, Department of Biomolecular Sciences from School of Pharmaceutical Sciences of Ribeirão Preto, University of São Paulo, Av. Do Café s/n CEP 14040-903, Ribeirão Preto -SP, Brazil; npelopes@fcfrp.usp.br

* Correspondence: thomschm@uni-muenster.de; Tel.: +49-251-83-33378 (T.J.S.)

***Biological assays* (methods previously reported in reference [23])**

All tests were performed using established and validated methods in the laboratories of Swiss TPH. Each compound was tested once against each parasite and the determination repeated in case of active compounds, (IC_{50} < 1 µg/mL) so that duplicate data were obtained in these cases. In such cases, the IC_{50} determination for cytotoxic activity was also repeated.

***In vitro* activity assay against *Trypanosoma brucei rhodesiense*.** Minimum Essential Medium (50 µL) supplemented with 25 mM HEPES, 1 g/L additional glucose, 1% MEM non-essential amino acids (100x), 0.2 mM 2-mercaptoethanol, 1mM Na-pyruvate, and 15% heat inactivated horse serum was disposed in 96-well microtiter plates. Serial compound dilutions of eleven 3-fold dilution steps covering a range from 100 to 0.002 µg/mL were prepared. Then, an aliquot containing $4x10^3$ bloodstream forms (trypomastigotes) of *T. b. rhodesiense* STIB 900 in 50 µL were added to each well and the plate incubated at 37 °C under 5 % CO_2 atmosphere for 70 h. After that, resazurin solution (12.5 mg in 100 mL water, 10 µL) was added to each well and the incubation continued for 2-4 h more. Then the plates were read using a Spectramax Gemini XS microplate fluorometer (Molecular Devices Cooperation, Sunnyvale, CA, USA) and excitation and emission wavelengths of 536 and 588 nm, respectively. Data were analyzed with the software Softmax Pro (Molecular Devices Cooperation, Sunnyvale, CA, USA), which calculated IC_{50} values by linear regression and 4-parameter logistic regression from the sigmoidal dose inhibition curves. Melarsoprol was used as positive control.

***In vitro* activity assay against *T. cruzi*.** Rat skeletal myoblasts (L-6 cells) were seeded in 96-well plates at 2000 cells/well in 100 µL RPMI 1640 medium with 10% FBS and 2 mM L-glutamine. After 24 h the medium was removed and replaced by 100 µL per well containing 5000 trypomastigote forms of *T. cruzi* Tulahuen strain C4 containing the β-galactosidase (Lac Z) gene. After 48 h the medium was removed from the wells and replaced by 100 µL fresh medium with or without a serial compound dilution (as mentioned for *T. brucei rhodesiense*). After 96 h of incubation the plates were inspected under an inverted microscope to assure growth of the controls and sterility. Then substrate CPRG/Nonidet (50 µL) was added to each well. The color reaction was developed within 2-6 h and the plate read at 540 nm. Data were analyzed as mentioned in the previous section. Benznidazole was used as positive control.

***In vitro* activity assay against *L. donovani* axenic amastigotes.** Amastigotes of *L. donovani* strain MHOM/ET/67/L82 were grown in axenic culture at 37 °C in SM medium at pH 5.4 supplemented with 10% heat-inactivated fetal bovine serum under a 5% CO_2 atmosphere. An aliquot containing 10^5 amastigotes from the axenic culture (100 µL) with or without a serial compound dilution (as specified before) were seeded in 96-well plates. After 70 h of incubation the plates were inspected under an inverted microscope to assure growth of the controls and sterile conditions. Then, resazurin solution (12.5 mg in 100 mL water, 10 µL) was added to each well and the plates incubated for another 2 h. Finally, the plates were read using the same conditions mentioned for *T. brucei rhodesiense*. Data were analyzed in the same way. Miltefosine was used as positive control.

***In vitro* activity assay against *P. falciparum*.** *In vitro* activity against erythrocytic stages of *P. falciparum* was determined using a ^3H-hypoxanthine incorporation assay, employing the drug sensitive NF54 strain (Schiphol Airport, The Netherlands). Compounds were diluted with medium as indicated above before added to parasite cultures incubated in RPMI 1640 medium without hypoxanthine, supplemented with HEPES (5.94 g/L), $NaHCO_3$ (2.1 g/L), neomycin (100 U/mL), AlbumaxR (5 g/L) and washed human red cells A+ at 2.5% hematocrit (0.3% parasitemia). Serial compound dilutions were prepared as before. The 96-well plates were incubated in a humidified atmosphere at 37 °C; 4% CO_2, 3% O_2, 93% N_2. After 48 h, 50 µL of 3H-hypoxanthine (0.5 µCi) was added to each well of the plate. The plates were incubated for a further 24 h under the same conditions. The plates were then harvested with a Betaplate™ cell harvester (Wallac, Zurich, Switzerland), and the red blood cells transferred onto a glass fiber filter then washed with distilled water. The dried filters were inserted into a plastic foil with 10 mL of scintillation fluid and counted in a Betaplate™ liquid scintillation counter (Wallac, Zurich, Switzerland). Data were treated as described above. Chloroquine and artemisinin were used as positive controls.

***In vitro* cytotoxicity assay with L-6 cells.** Assays were performed in 96-well plates. Each well was seeded with 100 µL of RPMI 1640 medium supplemented with 1% L-glutamine (200mM) and 10% fetal bovine serum, and 4000 L-6 cells. Serial compound dilutions prepared as before were used. After 70 h of incubation the plates were inspected under an inverted microscope to assure growth of the controls and sterile conditions. Resazurin solution (12.5 mg in 100 mL water, 10 µL) was then added to each well and the plates incubated for another 2 h. Finally, the plates were read, and the data were treated as mentioned before. Podophyllotoxin was used as positive control.

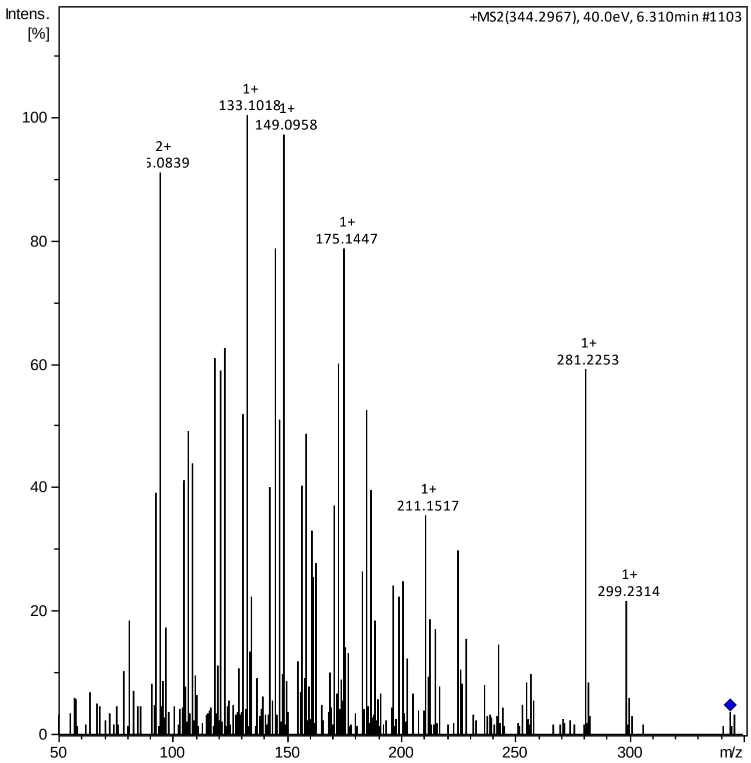

Intens.
[%]
+MS2(344.2967), 40.0eV, 6.310min #1103
1+
133.1018
1+
149.0958
2+
.0839
1+
175.1447
1+
281.2253
1+
211.1517
1+
299.2314
0
20
40
60
80
100
50
100
150
200
250
300
m/z

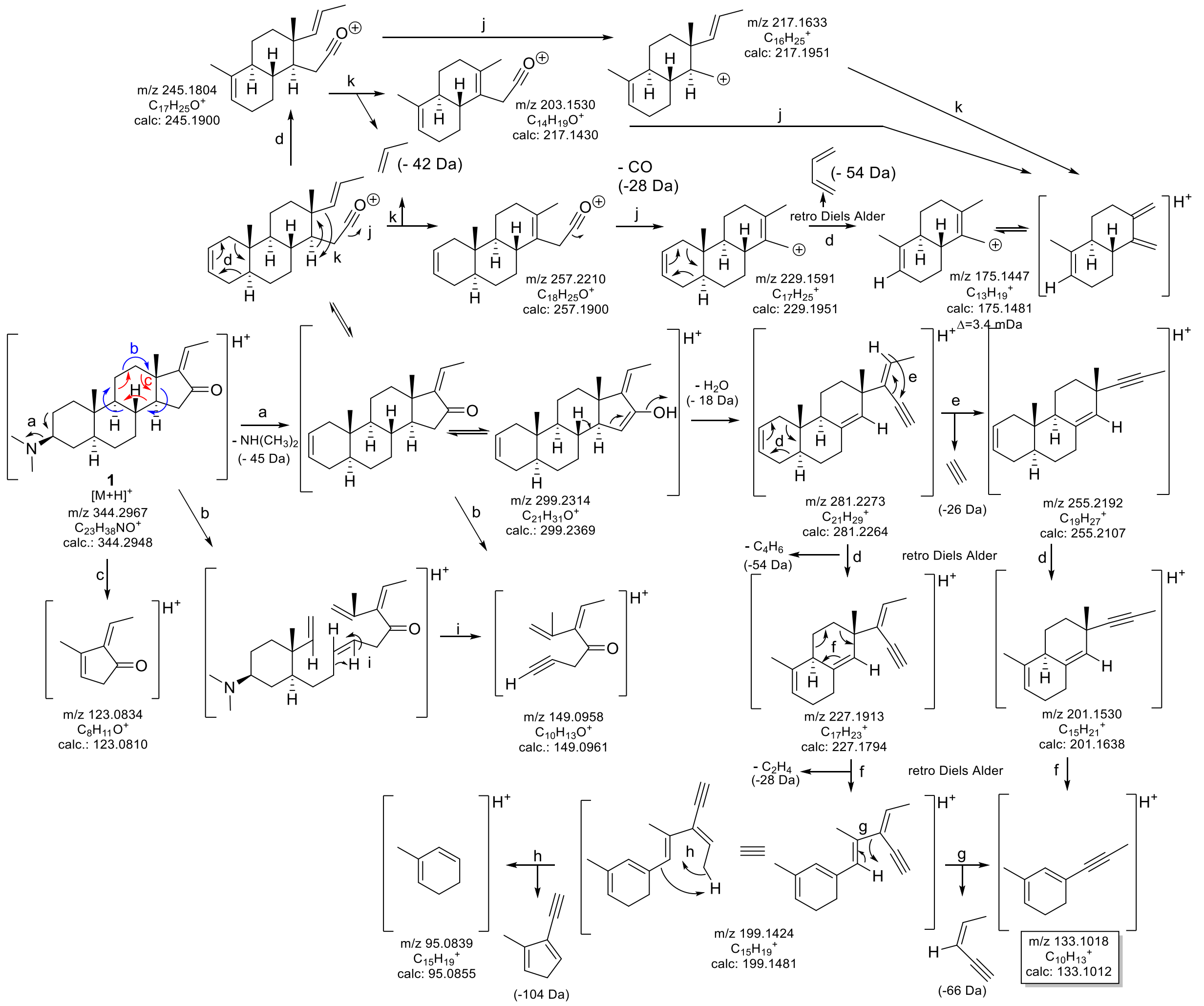

m/z 245.1804
C17H25O+
calc: 245.1900
j
k
m/z 203.1530
C14H19O+
calc: 217.1430
m/z 217.1633
C16H25+
calc: 217.1951
(- 42 Da)
d
- CO
(-28 Da)
(- 54 Da)
retro Diels Alder
m/z 257.2210
C18H25O+
calc: 257.1900
m/z 229.1591
C17H25+
calc: 229.1951
m/z 175.1447
C13H19+
calc: 175.1481
Δ=3.4 mDa
H+
1
[M+H]+
m/z 344.2967
C23H38NO+
calc.: 344.2948
a
- NH(CH3)2
(- 45 Da)
OH
m/z 299.2314
C21H31O+
calc.: 299.2369
- H2O
(- 18 Da)
m/z 281.2273
C21H29+
calc: 281.2264
e
(-26 Da)
m/z 255.2192
C19H27+
calc: 255.2107
b
c
- C4H6
(-54 Da)
m/z 123.0834
C8H11O+
calc.: 123.0810
i
m/z 149.0958
C10H13O+
calc.: 149.0961
f
m/z 227.1913
C17H23+
calc: 227.1794
m/z 201.1530
C15H21+
calc: 201.1638
- C2H4
(-28 Da)
g
h
m/z 95.0839
C15H19+
calc: 95.0855
(-104 Da)
m/z 199.1424
C15H19+
calc: 199.1481
(-66 Da)
m/z 133.1018
C10H13+
calc: 133.1012

Figure S1. (+)ESI MS^2 spectrum and fragmentation pathway for the $[M+H]^+$ ion of compound **1** (E/Z Salignone [12,13]; shown is the Z-form) at *m/z* 344.2967. Spectrum taken from fraction A5D.

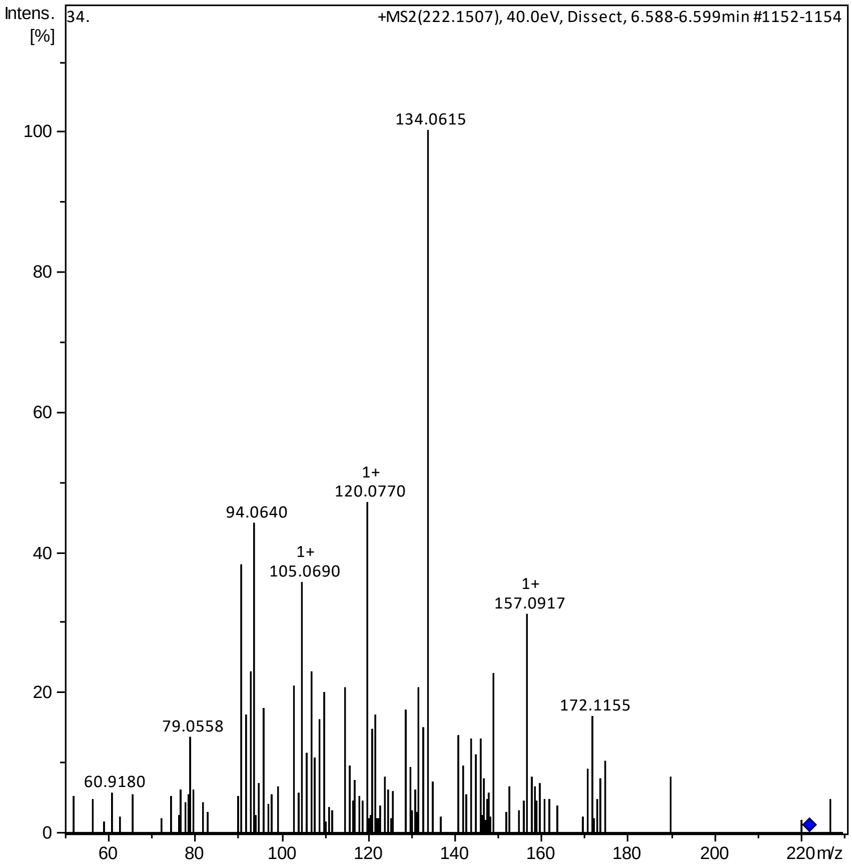

Figure S2. (+)ESI MS^2 spectrum and fragmentation pathway for the $[M+H]^+$ ion of compound **2** at *m/z* 222.1507. Spectrum taken from fraction 2C3. Note that this compound is not an aminosteroid. No further characterization was possible and the spectrum is presented only for completeness.

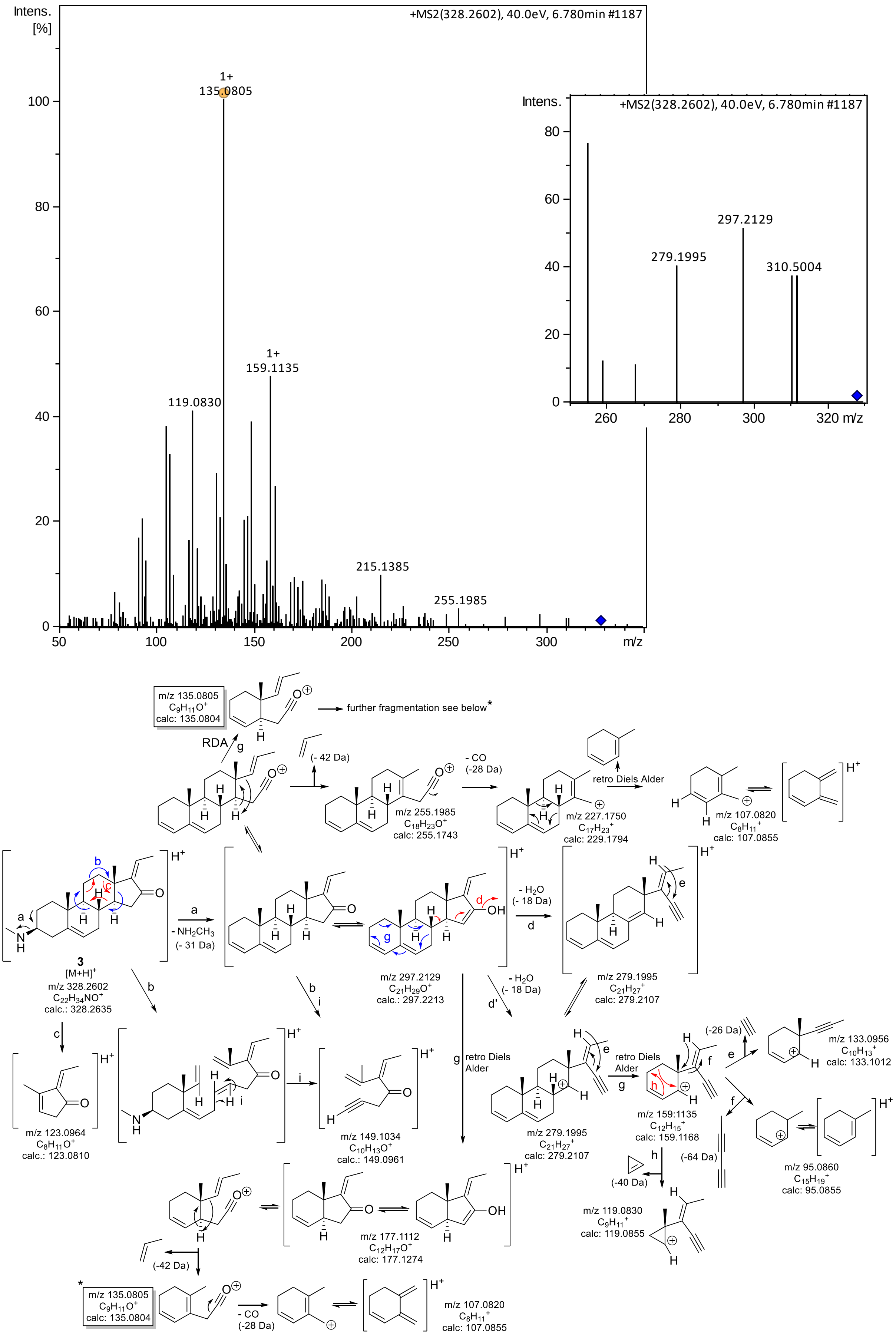

Figure S3. (+)ESI MS^2 spectrum and fragmentation pathway for the $[M+H]^+$ ion of compound **3** (3β-Methylamino-16-oxo-5,17(20)-cis/trans-pregnadiene [12]; shown is the *Z*-(*cis*)-form) at *m/z* 328.2602. (Spectrum taken from fraction 2D).

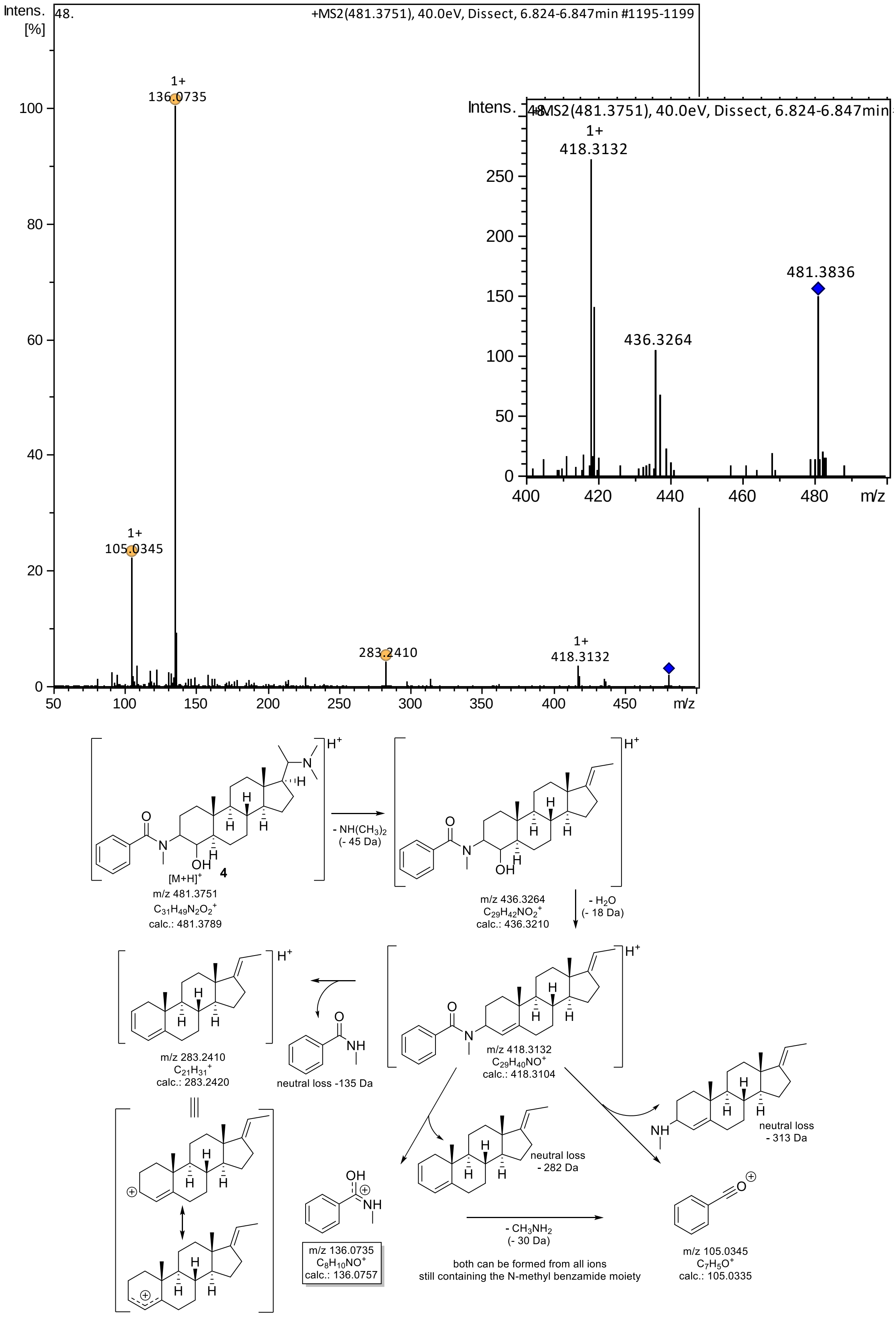

Figure S4. (+)ESI MS^2 spectrum (top: full mass range, bottom: magnified high *m/z* region) and fragmentation pathway for the $[M+H]^+$ ion of compound **4** at *m/z* 481.3751 (note that the OH group was postulated at C-4 in analogy to the known acetate **6**, compare with Figure S5). (Spectrum taken from fraction 3C).

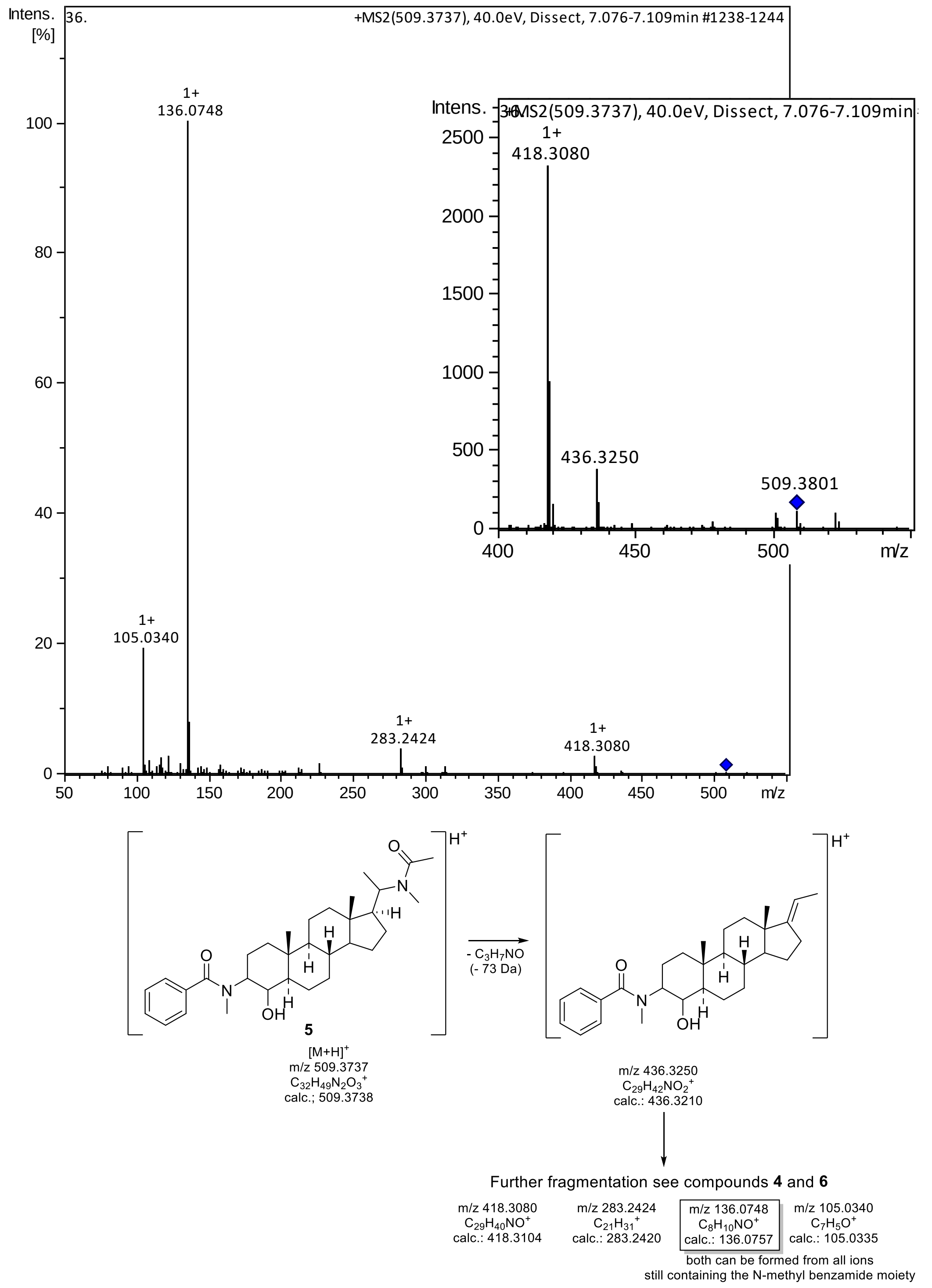

Figure S5. (+)ESI MS^2 spectrum and fragmentation pathway for the $[M+H]^+$ ion of compound **5** at *m/z* 509.3737. (Spectrum taken from fraction A5H).

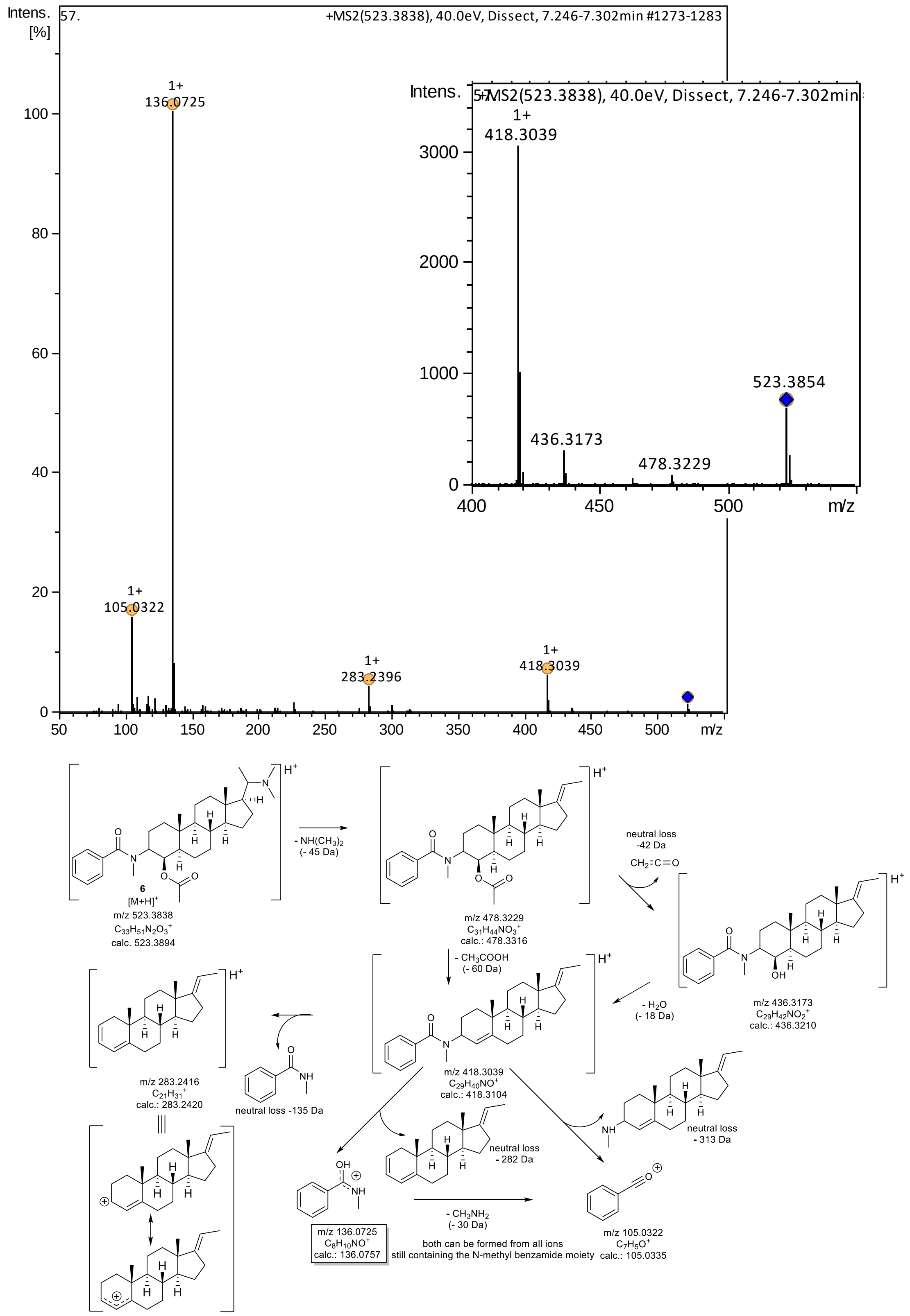

Figure S6. (+)ESI MS^2 spectrum (top: full mass range, bottom: magnified high *m/z* region) and fragmentation pathway for the $[M+H]^+$ ion of compound **6** at *m/z* 523.3838 (Pachysandrin A [14]; compare with Figure S3). (Spectrum taken from fraction A).

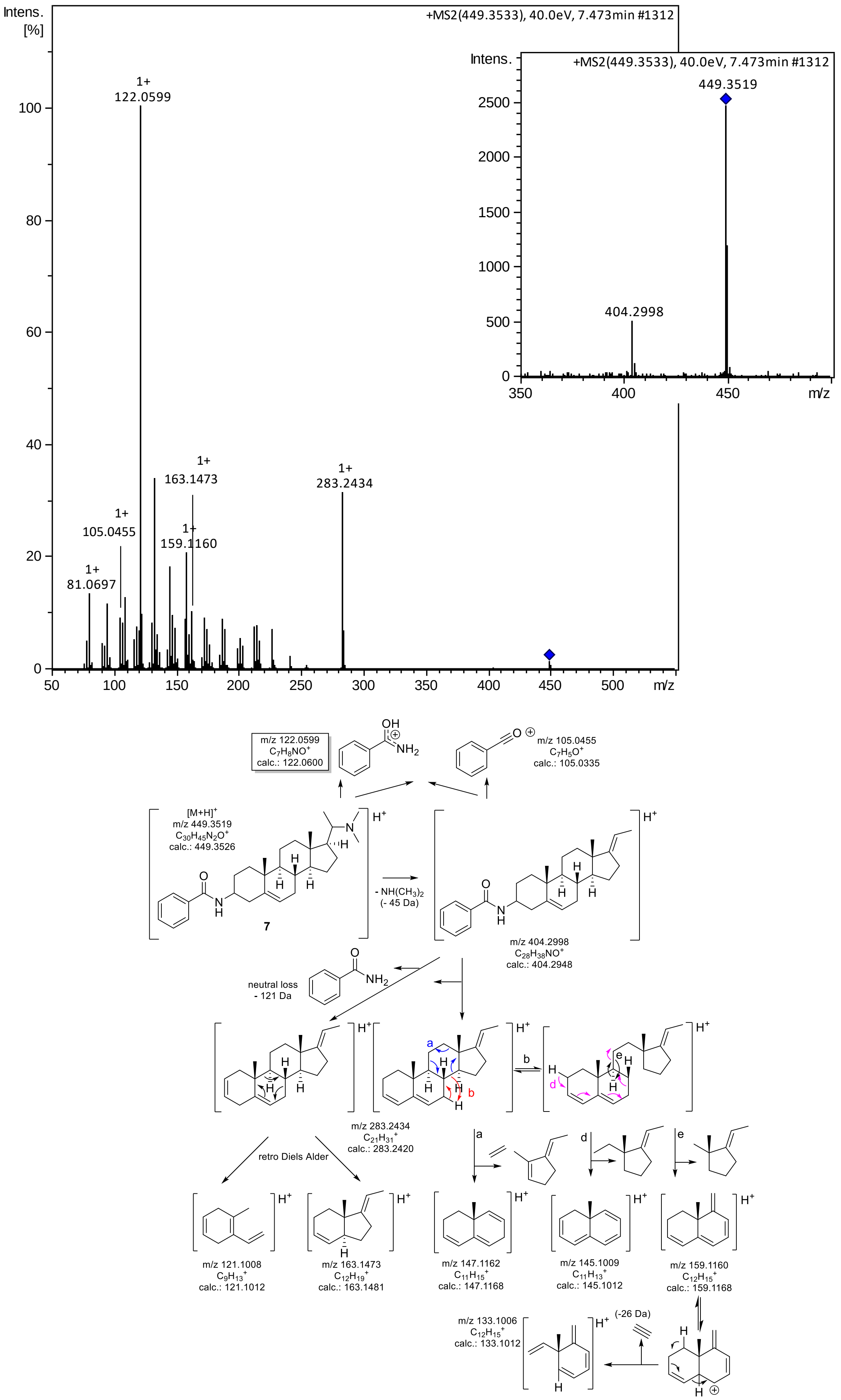

Figure S7. (+)ESI MS^2 spectrum and fragmentation pathway for the $[M+H]^+$ ion of compound **7.** (Spectrum taken from fraction A5K).

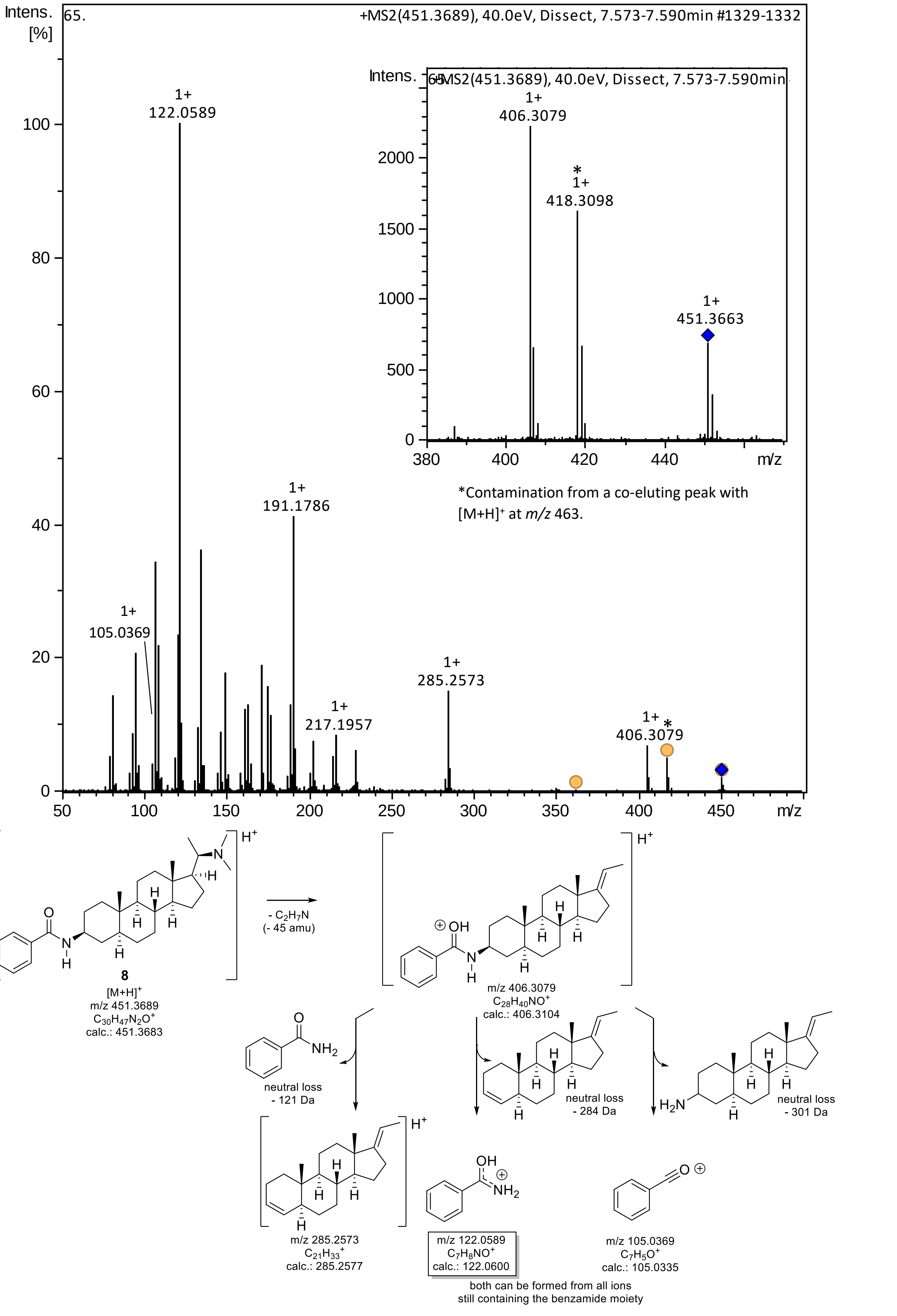

Figure S8. (+)ESI MS^2 spectrum and fragmentation pathway for the $[M+H]^+$ ion of compound **8** (Epipachysamine D [9, 10] at *m/z* 451.3689. (Spectrum taken from fraction A5).

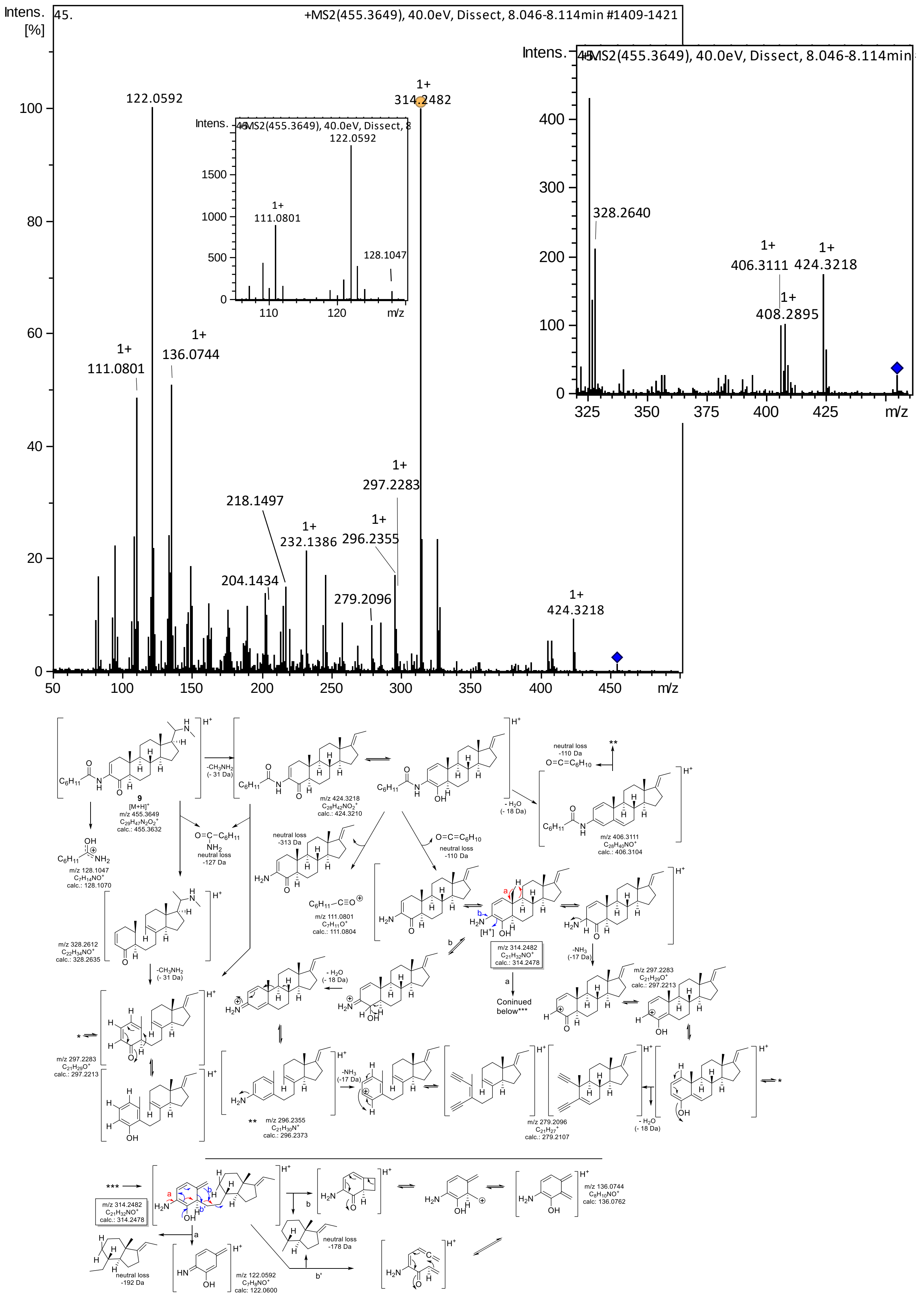

Figure S9. (+)ESI MS[2] spectrum and fragmentation pathway for the $[M+H]^+$ ion of compound **9** at *m/z* 455.3649. (Spectrum taken from fraction A5D).

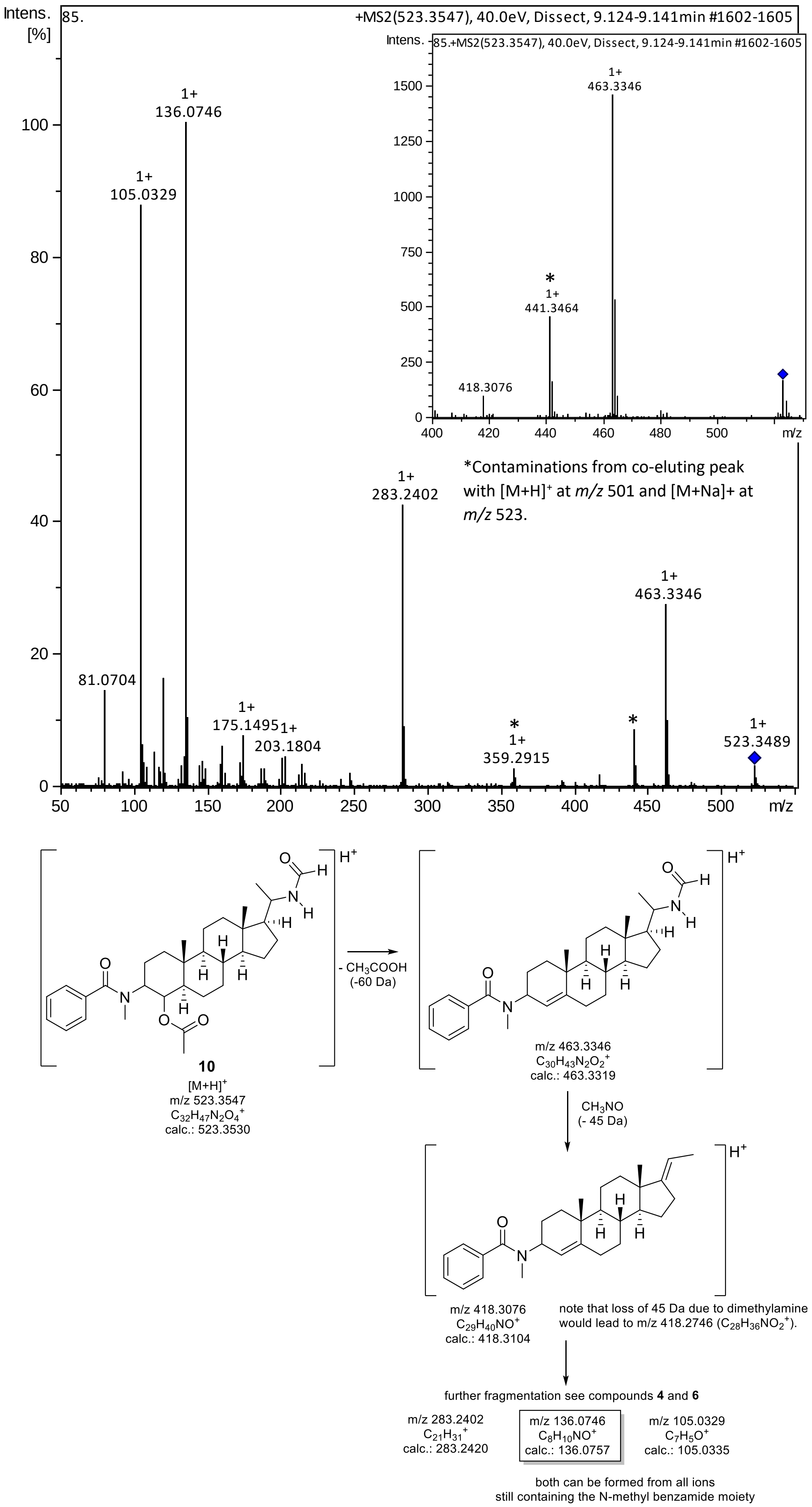

Figure S10. (+)ESI MS^2 spectrum and fragmentation pathway for the $[M+H]^+$ ion of compound **10** at *m/z* 523.3547(note that the acetoxy group could also be at C-2). (Spectrum taken from fraction A5P).

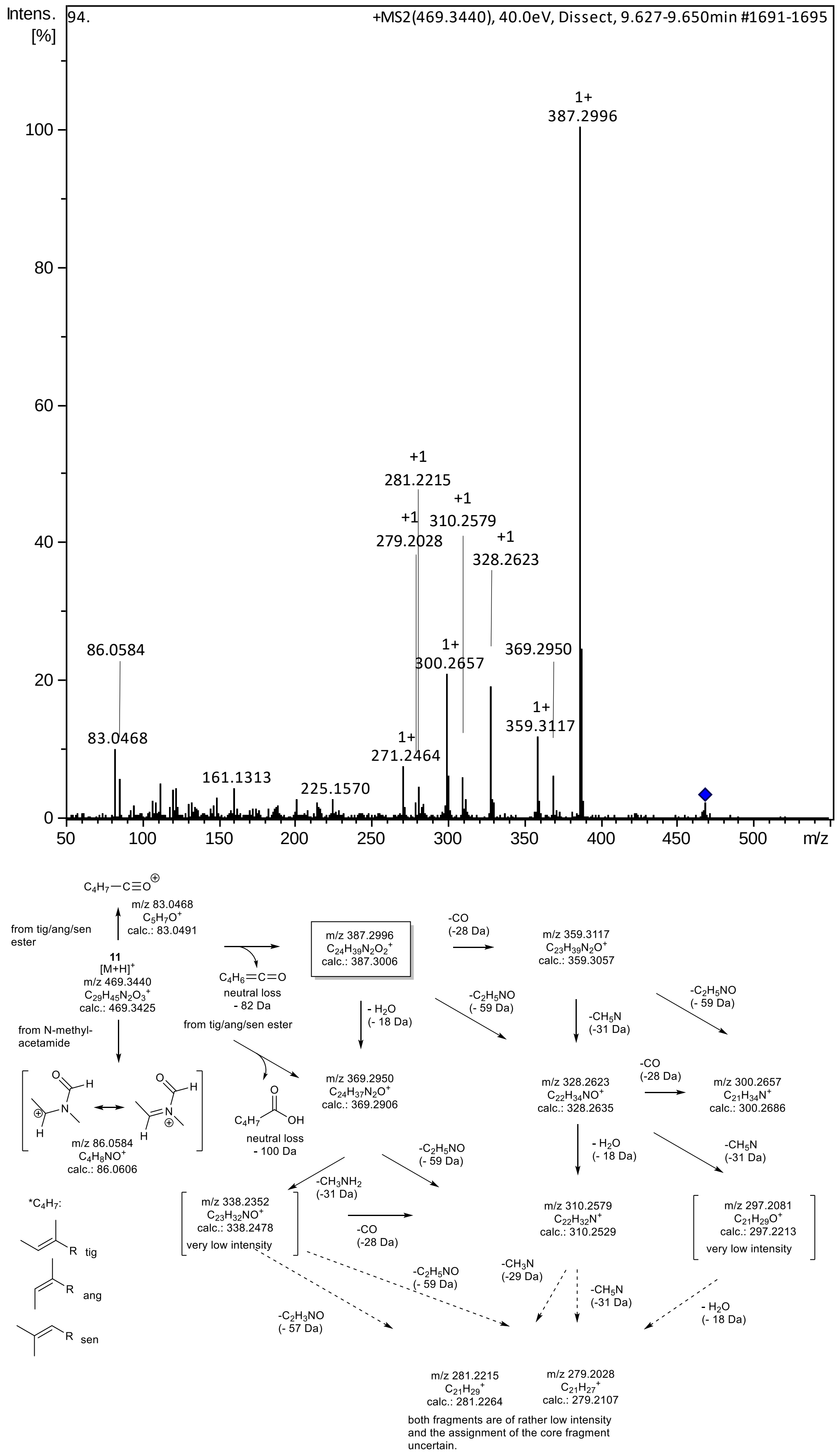

Figure S11. (+)ESI MS^2 spectrum and fragmentation pathway for the $[M+H]^+$ ion of compound **11** at *m/z* 469.3440. (Spectrum taken from fraction A5P).

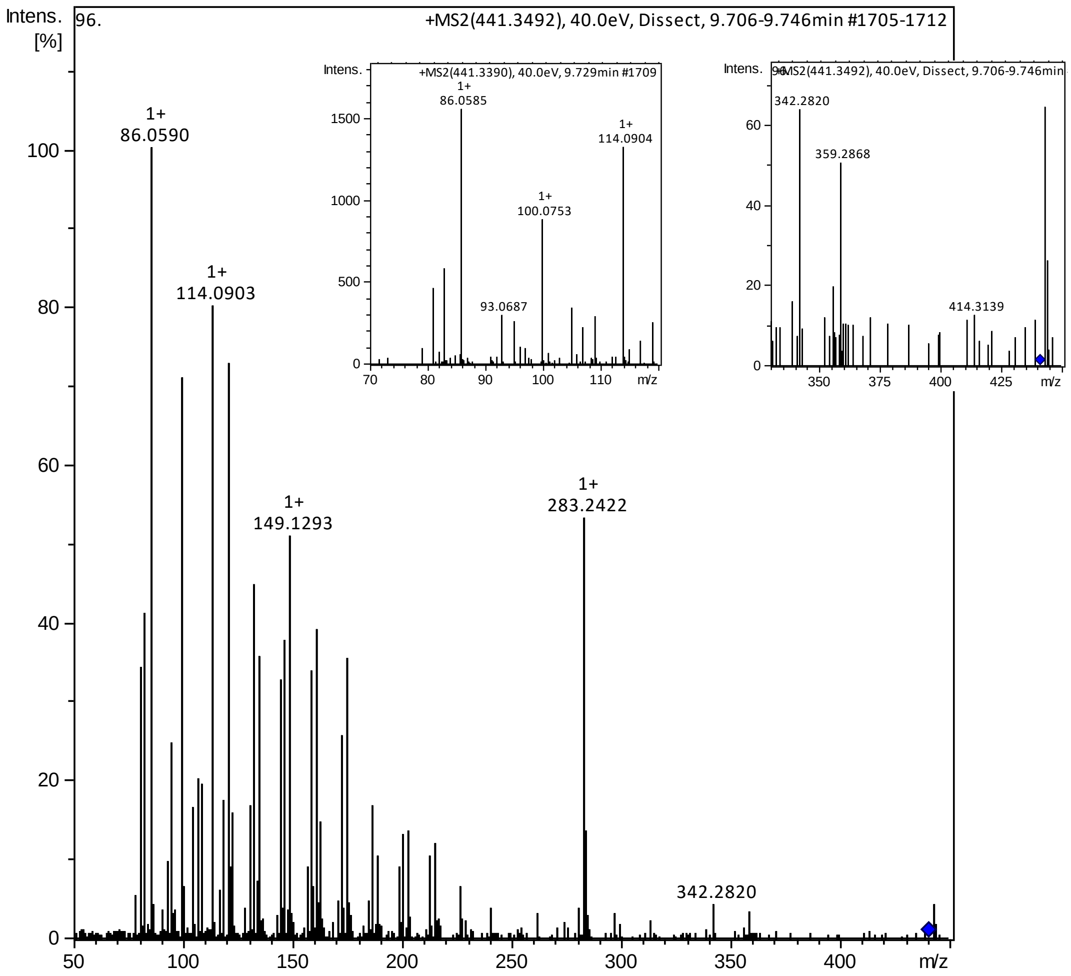

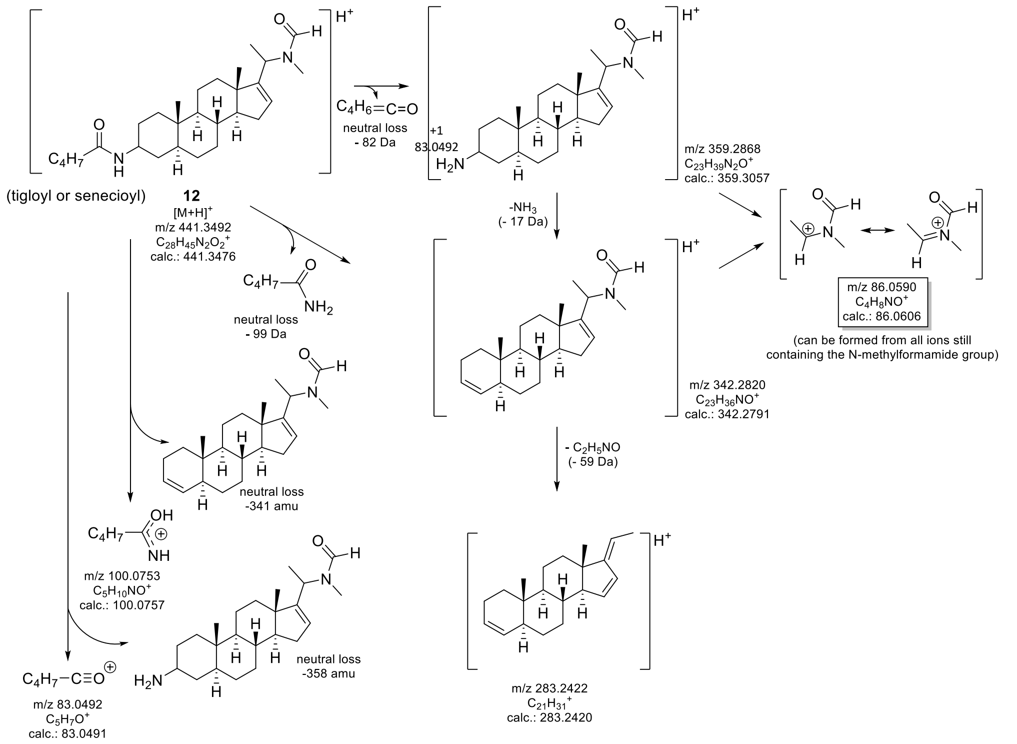

Figure S12. (+)ESI MS^2 spectrum and fragmentation pathway for the $[M+H]^+$ ion of compound **12** at *m/z* 441.3492. (Spectrum taken from fraction A5P).

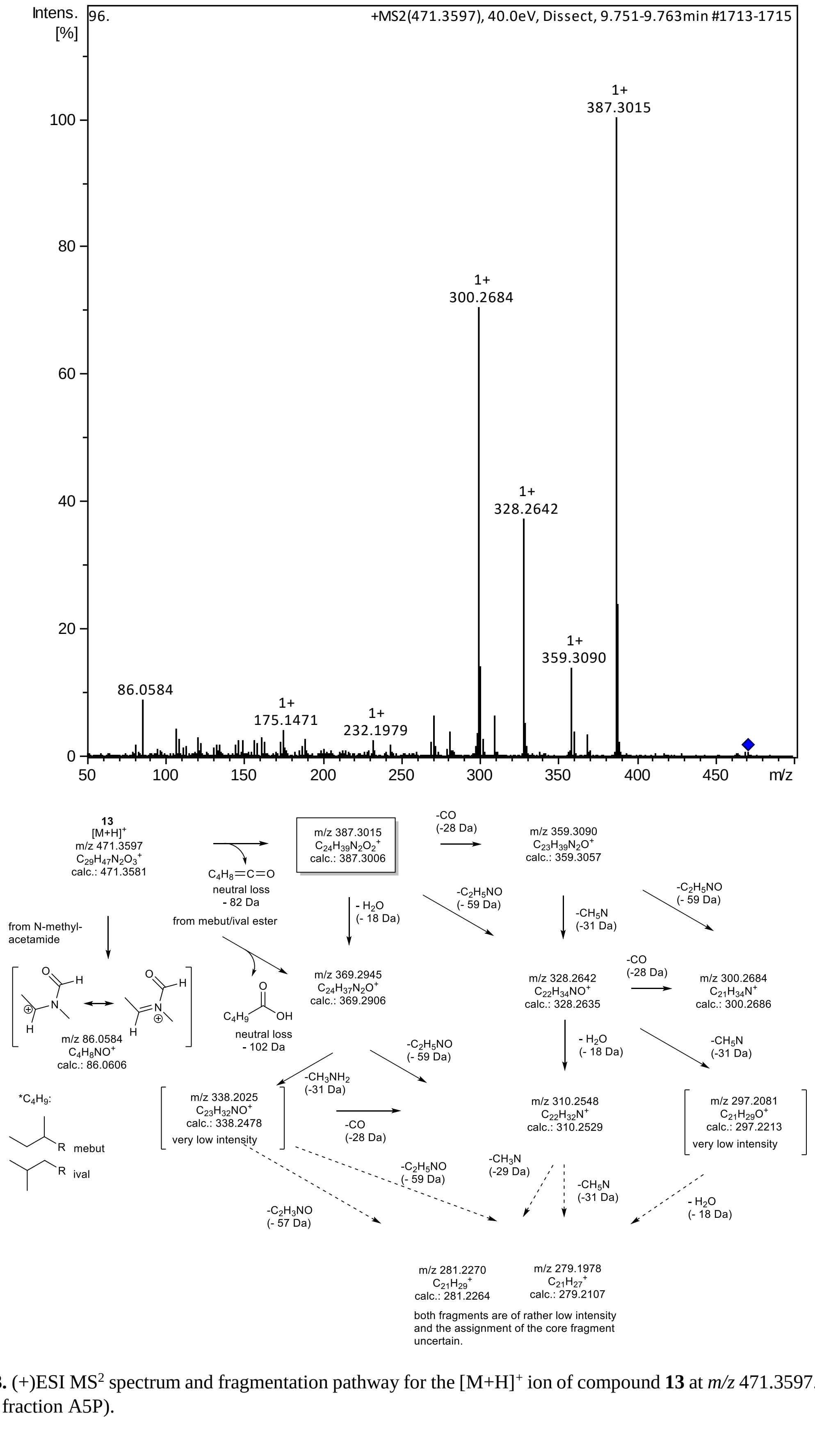

Figure S13. (+)ESI MS^2 spectrum and fragmentation pathway for the $[M+H]^+$ ion of compound **13** at *m/z* 471.3597. (Spectrum taken from fraction A5P).

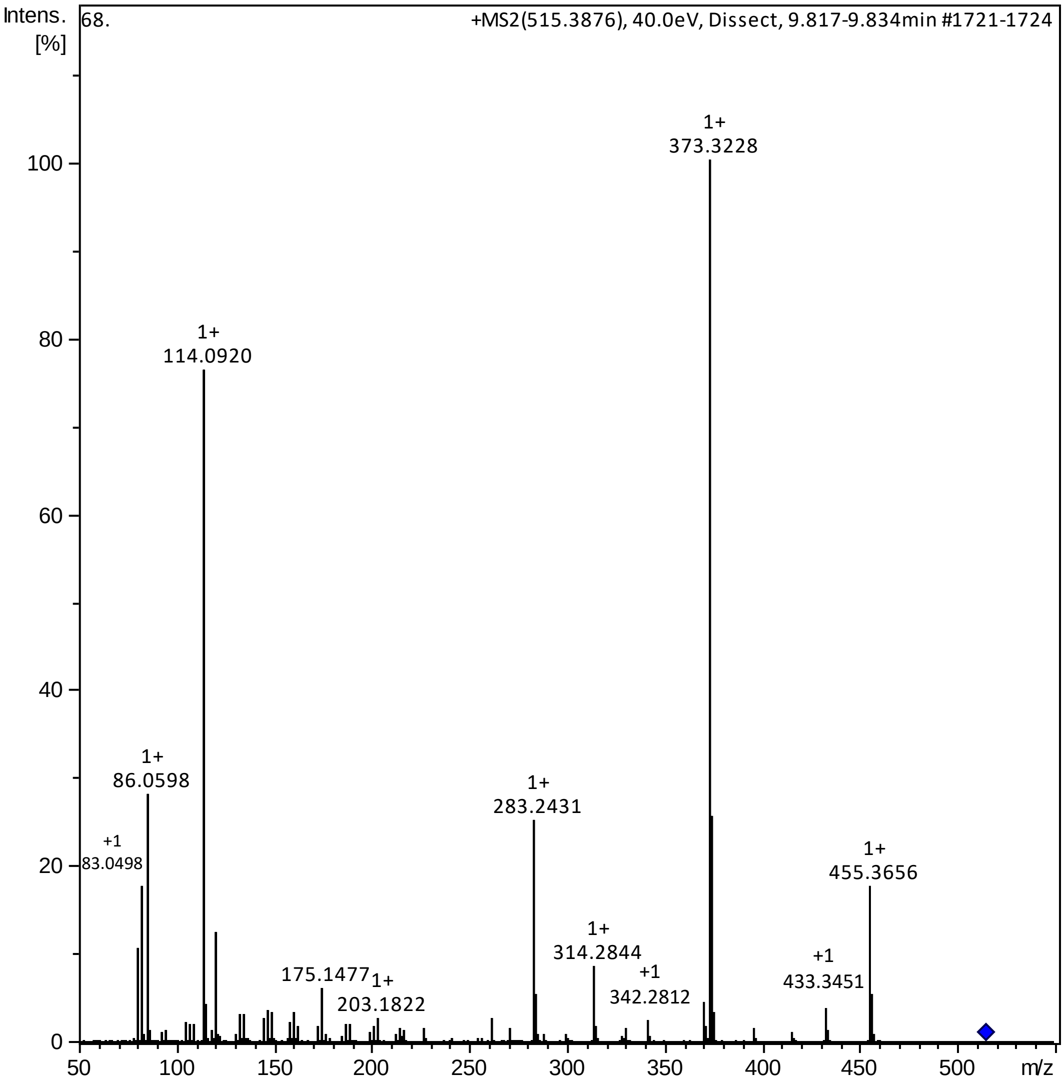

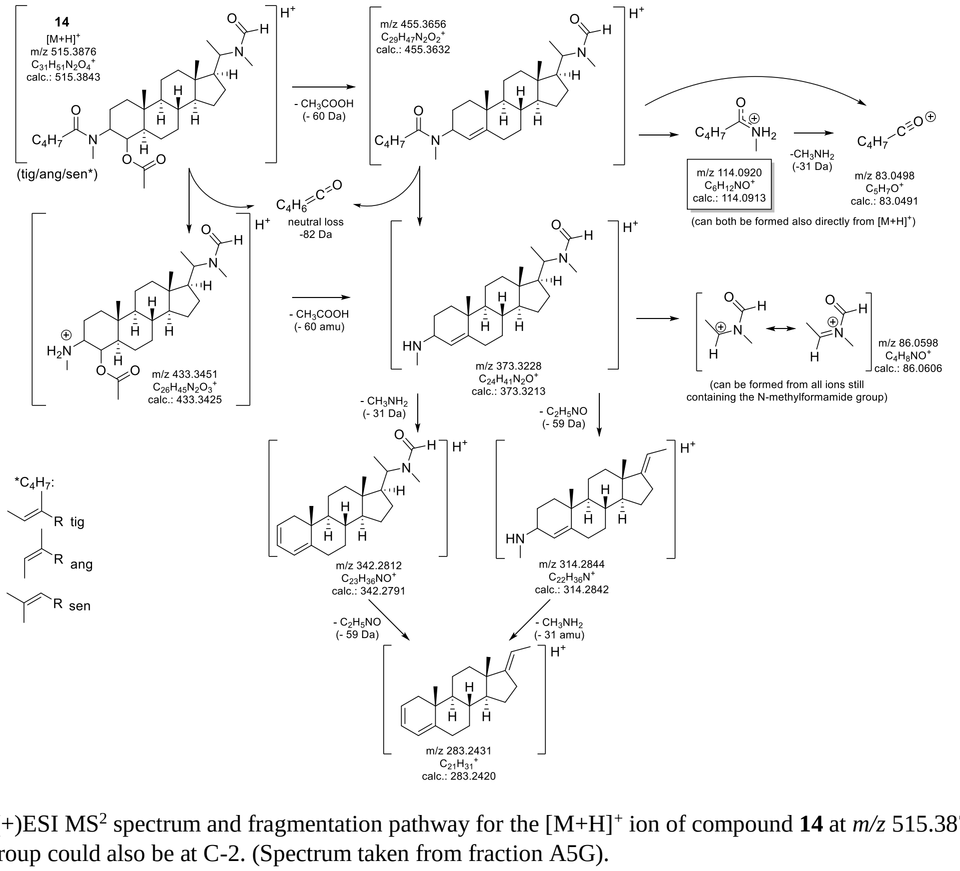

Figure S14. (+)ESI MS^2 spectrum and fragmentation pathway for the $[M+H]^+$ ion of compound **14** at *m/z* 515.3876.Note that the acetoxy group could also be at C-2. (Spectrum taken from fraction A5G).

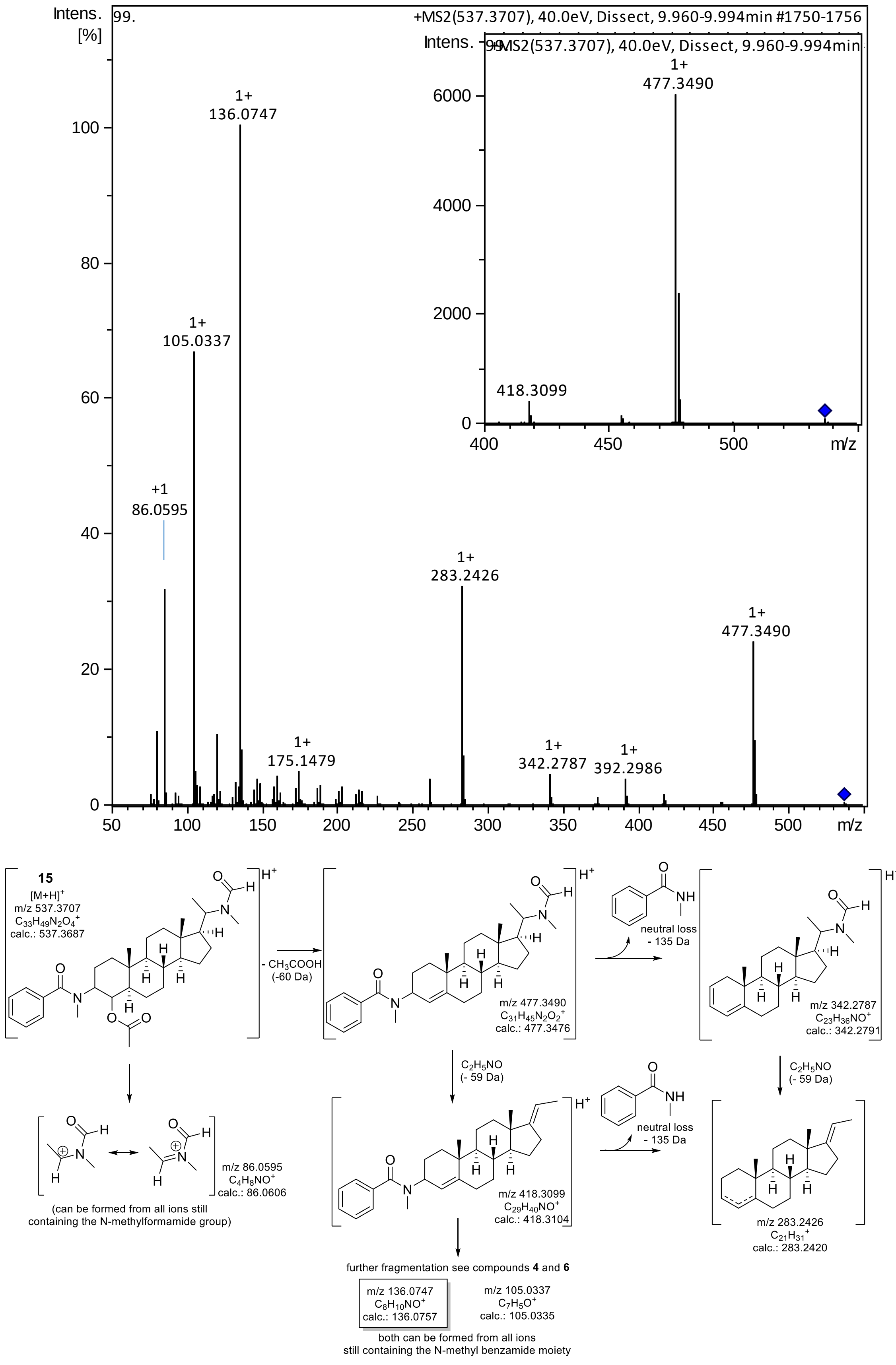

Figure S15. (+)ESI MS^2 spectrum and fragmentation pathway for the $[M+H]^+$ ion of compound **15** at *m/z* 537.3707 (note that the acetate moiety could also be at C-2).(Spectrum taken from fraction A5P).

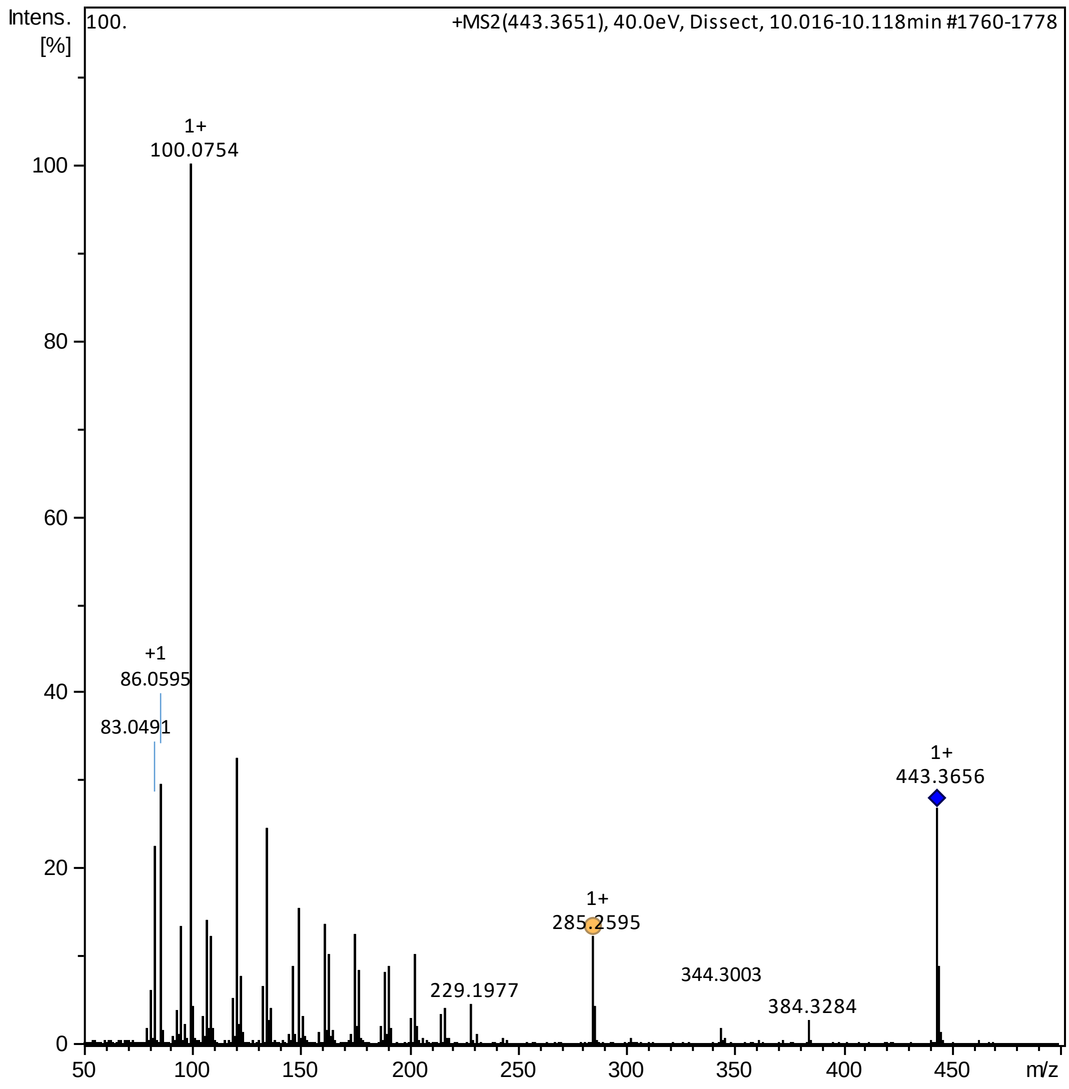

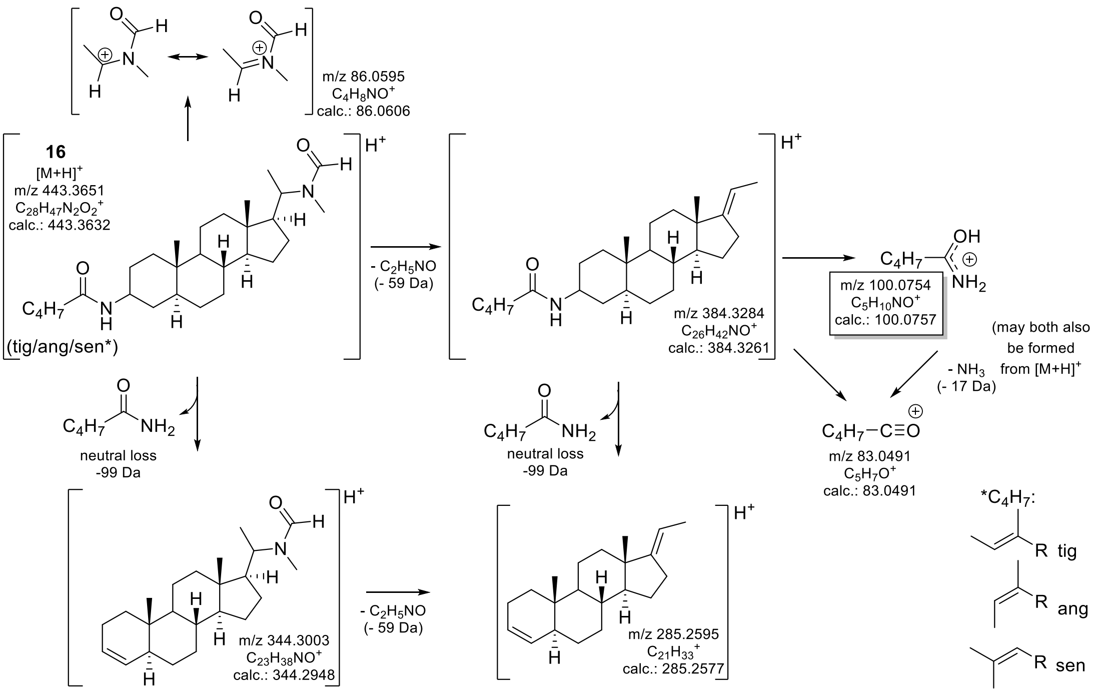

Figure S16. (+)ESI MS^2 spectrum and fragmentation pathway for the $[M+H]^+$ ion of compound **16** at *m/z* 443.3651. (Spectrum taken from fraction A5P).

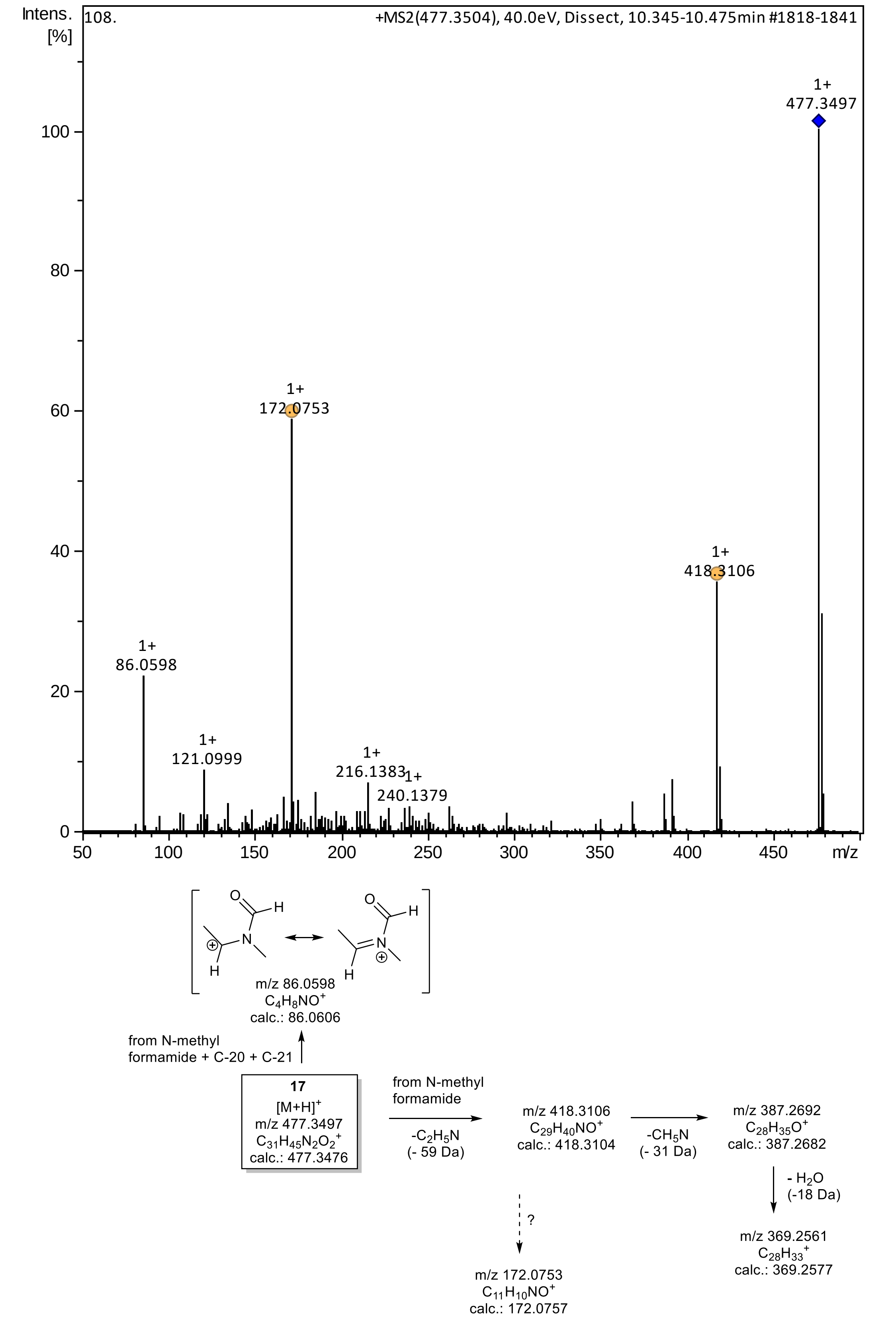

Figure S17. (+)ESI MS^2 spectrum and fragmentation pathway for the $[M+H]^+$ ion of compound **17** at *m/z* 477.3504. (Spectrum taken from fraction A5P).

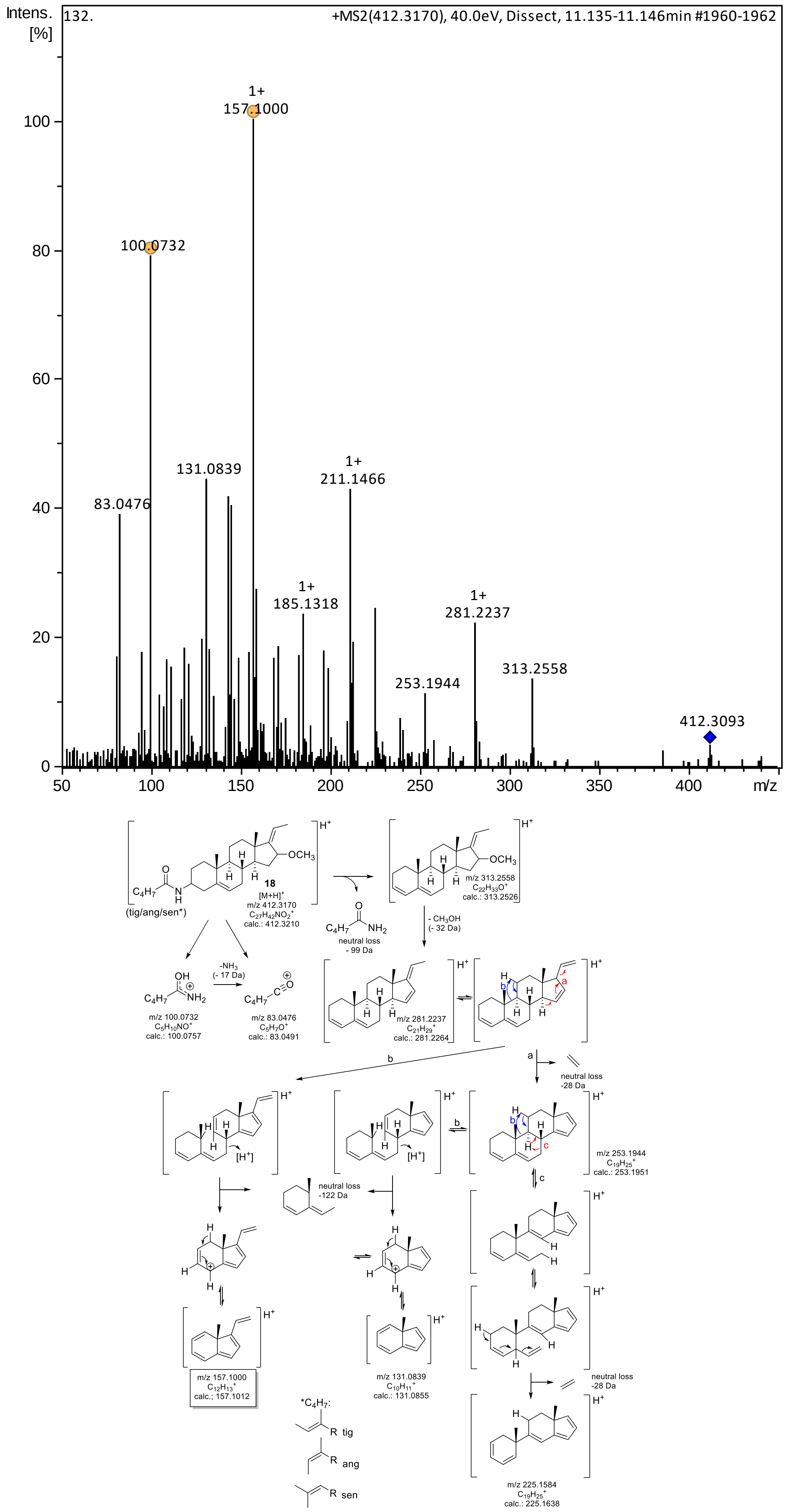

Figure S18. (+)ESI MS^2 spectrum and fragmentation pathway for the $[M+H]^+$ ion of compound **18** at *m/z* 412.3170. (Spectrum taken from fraction A).
